# Photocatalytic C-N coupling from stable and transient intermediates for gram-scale acetamide synthesis

**DOI:** 10.1038/s41467-025-58840-0

**Published:** 2025-04-15

**Authors:** Xin Li, Weiping Yang, Junping Yue, Jieyuan Li, Shujie Shen, Ruimin Chen, Jielin Wang, Huimin Dan, Dagang Yu, Fan Dong

**Affiliations:** 1 Research Center for Carbon-Neutral Environmental & Energy Technology, Institute of Fundamental and Frontier Sciences, University of Electronic Science and Technology of China, Chengdu, 611731 China; 2 Key Laboratory of Green Chemistry & Technology of Ministry of Education, College of Chemistry, Sichuan University, Chengdu, 610064 China

**Keywords:** Photocatalysis, Chemical synthesis, Materials for energy and catalysis

## Abstract

Electro/photocatalytic C-N coupling acts as a key build-block to the next generation of chemicals like amides for wide applications in energy, pharmaceuticals and chemical industries. However, the uncontrolled intermediates coupling challenges the efficient amide production regarding yield or selectivity. Here we propose a photocatalytic radical addition route, where the fundamental active species, including oxygen and photogenerated electron-hole pairs, are regulated for selective intermediates generation and efficient acetamide synthesis from mild co-oxidation of CH_3_CH_2_OH and NH_3_. Sufficient CH_3_CH_2_OH is provided to accumulate the stable intermediate (CH_3_CHO). Meanwhile, the limited NH_3_ concentration ensures the controllable generation and fast addition of the transient radical (^●^NH_2_) on CH_3_CHO. Through the directed coupling of stable-transient intermediates, the acetamide synthesis rate is pushed forward to a hundred-mmol level (105.61 ± 4.86 mmol·g_cat_^−1^·h^−1^) with a selectivity of 99.17% ± 0.39%, reaching a gram-scale yield (1.82 g) of acetamide. These results illuminate valuable opportunities for the photocatalysis-driven synthetic industry.

## Abstract (web summary)

This study explores N–heterocyclic carbene copper complexes toward selective electrocatalytic reduction of acetylene to ethylene. The electron–rich copper sites were found to facilitate acetylene adsorption and ethylene desorption and achieved high activity and selectivity for ethylene production.

## Introduction

Amides and their derivatives are some of the most important essential chemicals in the food, pharmacy, and chemical industries^1–3^. The efficient and economical synthesis of amides contributes greatly to the sustainable development of human society. However, industrial amide synthesis still relies on coupling the C- and N-containing small molecules under harsh conditions. Taking acetamide synthesis as a typical case, CH_3_COOH and NH_3_ are fed for coupling with the assistance of dehydrators and catalysts at a high temperature of 150–180 °C, which consumes massive fossil fuels and leads to huge carbon emissions^4–6^. Hence, substantial research efforts have been devoted to developing alternative routes for amide synthesis under ambient conditions. Among them, the electrochemical method for co-reducing CO_2_/CO and N_2_/NO_x_^−^, has recently attracted increased interest, in which obvious advances have been achieved for electrochemical amide synthesis from C-N coupling^7–11^. However, the corresponding synthesis rate and selectivity (or Faraday efficiency, FE) are still restrained to low levels^12–14^. Specifically, under lower bias voltage, the activation of the reactant molecules is insufficient for their catalytic transformation, resulting in a limited synthesis rate^15–17^. On the contrary, the separate deep reduction of C- and N-substance is inevitable when the electric energy supply is excess, making it rather difficult to form the C-N bond selectively, thus failing to increase the selectivity of amides^18–20^. Hence, a mild redox ability, contributed by heterogeneous photocatalysis, may be an alternative approach for the efficient synthesis of amides from selective C-N coupling^21–24^.

To this end, the precise regulation and selective generation of the key intermediates for C-N coupling, including carbonyl (-C=O) and amino (-NH_2_) species, are imperative. However, these species are located in their intermediated valence states, respectively. Therefore, a precisely tailored mild redox reaction route should be constructed for their delicate preparation^25–27^. Otherwise, side reactions of the per-oxidation/per-reduction are unavoidable under a rough redox driving force, which results in decreased selectivity for amide synthesis. Moreover, it is well-acknowledged that a low reaction rate is expected by starting the coupling reactions with two stable intermediates due to their low chemical activity^28–30^. In contrast, the coupling possibility between two transient intermediates, such as reactive radicals, is severely limited since multiple disordered side reactions proceed in their short lifetimes^29,31,32^. That is, a complementary couple of stable-transient intermediates is required to endow the C-N coupling reaction with both optimum production rate and selectivity synergistically. By making use of the mild redox ability of photocatalysis and optimizing the coupled intermediates, it is anticipated that a meticulously designed photocatalytic reaction system can be developed for efficient synthesis of amides.

**Fig. 1:**
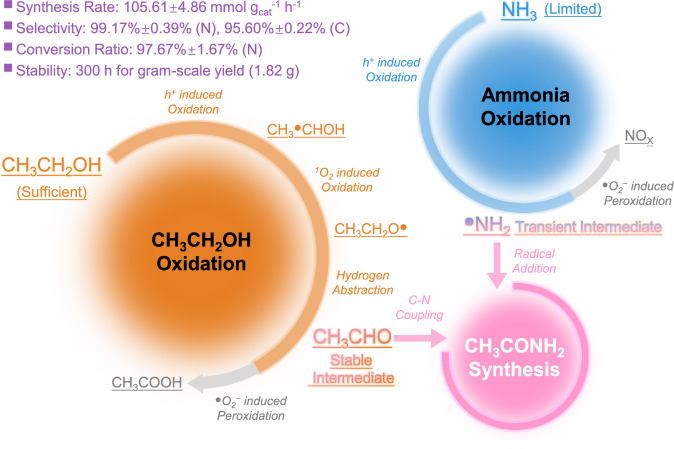
Schematic diagram. Illustration for the coupling of stable and transient intermediates for acetamide photosynthesis.

Herein, we propose a photocatalytic radical addition route for acetamide synthesis (Fig. 1), using CH_3_CH_2_OH and NH_3_ as the C- and N-source, respectively. Under the mild photocatalytic redox driving force, the fundamental active species, including O_2_ and electron (e^−^)-hole (h^+^) pairs, are regulated for selective co-oxidation of CH_3_CH_2_OH and NH_3_. Specifically, sufficient CH_3_CH_2_OH is provided to accumulate CH_3_CHO as the stable intermediate. Meanwhile, the limited provision of NH_3_ ensures the controllable generation of ^●^NH_2_ as the transient intermediate, in which the fast addition of ^●^NH_2_ radical into CH_3_CHO is subsequently achieved. The oxidative ability of this reaction system is tailored by quantified O_2_ activation, which guarantees considerable CH_3_CONH_2_ selectivity and impedes the peroxidation of CH_3_CHO and ^●^NH_2_ intermediates. The acetamide synthesis rate is pushed forward to a hundred-mmol level (105.61 ± 4.86 mmol g_cat_^−1^ h^−1^) with superior selectivity (99.17% ± 0.39%), significantly exceeding most of the other catalytic routes. The photocatalysis system can be stably and continuously operated for 300 h, during which the acetamide yield reaches the gram scale (1.82 g). A comprehensive mechanism investigation is conducted to reveal the actual coupling coordinates and key intermediates, in which direct evidence has been provided based on the iso-type (D and ^15^N) labeled in situ EPR, HR-MS, NMR, and in situ ATR-FTIR. It is clarified that the efficient coupling of the stable and transient intermediates is the decisive factor for achieving optimum catalytic activity. We anticipate the current demonstration to be a starting point for realizing the efficient production of amide compounds with a simple but practical heterogeneous photocatalysis scheme, thus increasing the possibility of the science and industry revolution driven by solar light.

## Results and discussion

### Reaction system design and efficiency evaluation

The commercial TiO_2_ (P25) is applied as a representative photocatalyst to develop the photoredox route for C-N coupling, which only absorbs the photon and forms the active e^−^ and h^+^ in this system, excluding the specificity of other modified photocatalysts and establishes a general pattern for the superiority of the photocatalytic amides synthesis scheme. Other common photocatalysts are also investigated in the C-N coupling reaction, which processes similar properties, showing the universality of photocatalysts in C-N coupling (Supplementary Fig. 4 and Supplementary Note 3). At first, various photocatalytic routes, including co-oxidation, co-reduction, and combined redox reactions, are screened to examine the practicability of amide synthesis (Table 1). It is identified that the amide bonds can be constructed through the coupling of various C_1_/C_2_ and N_1_ substances, in which significant promotion of the synthesis efficiency is observed in the reaction route of CH_3_CH_2_OH and NH_3_ co-oxidation for CH_3_CONH_2_ production (109.24 μmol h^−1^), remarkably exceeding the other photocatalysis routes. Therefore, CH_3_CH_2_OH and NH_3_ reactants are assigned as the respective C- and N-source for the comprehensive efficiency evaluation and mechanism investigations.

**Table. 1:** List of screening experiments of different photocatalytic redox routes for amide synthesis

C-source	N-source	Product	Synthesis rate (μmol h^−1^)^a^
CH_3_OH	NH_3_	HCONH_2_	51.99
CH_3_OH	NO_2_^−^	HCONH_2_	40.44
CH_3_OH	NO_3_^−^	HCONH_2_	41.21
HCOOH	NH_3_	HCONH_2_	0.32
HCOOH	NO_2_^−^	HCONH_2_	trace
HCOOH	NO_3_^−^	HCONH_2_	0.57
CH_3_CH_2_OH	NH_3_	CH_3_CONH_2_	109.24
CH_3_CH_2_OH	NO_2_^−^	CH_3_CONH_2_	23.41
CH_3_CH_2_OH	NO_3_^−^	CH_3_CONH_2_	16.01
CH_3_COOH	NH_3_	CH_3_CONH_2_	trace
CH_3_COONa	NH_3_	CH_3_CONH_2_	trace
CH_3_COOH	NO_2_^−^	CH_3_CONH_2_	6.55
CH_3_COOH	NO_3_^−^	CH_3_CONH_2_	trace

^a^The respective standard curves of HCONH_2_, CH_3_CONH_2_, and CO(NH_2_)_2_ (Supplementary Figs. 1–3) are provided in the Supplementary Information.

**Fig. 2:**
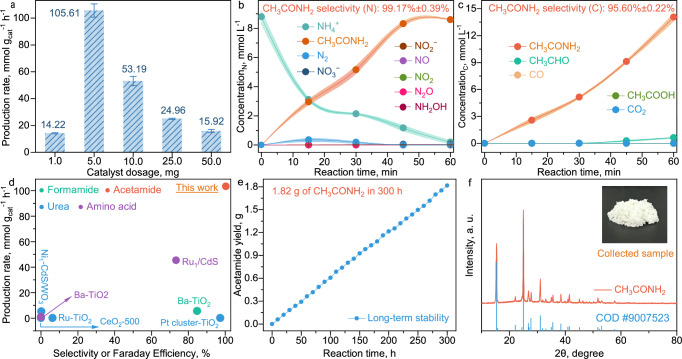
Efficiency evaluation for acetamide photosynthesis. **a** catalyst dosage-dependent unit production rate; CH_3_CONH_2_ selectivity evaluation regarding the N-(**b**) and C-sources (**c**), respectively; **d** Efficiency comparison between different catalytic routes for photocatalytic C-N coupling, including the targets of production rate and selectivity, the corresponding research works are listed and cited in  Supplementary Information (Supplementary Table 3)^54–60^; **e** Long-term stability test. **f** XRD pattern and the image (inset) of the collected CH_3_CONH_2_ sample with rotary evaporation after the long-term stability test. The respective standard curves for detecting the reaction species using ion chromatography (IC, NH_4_^+^, NO_2_^−^, NO_3_^−^, CH_3_COOH, and NH_2_OH, Supplementary Figs. 31–35) are provided in  Supplementary Information. The error bars in (**a**–**c**) were drawn based on the calculated standard error of two parallel tests.

After the optimization of the reaction parameters, including NH_3_ concentration, CH_3_CH_2_OH proportion, O_2_ proportion (in Ar), and catalyst dosage (Supplementary Figs. 5–9 and Supplementary Note 16), a high CH_3_CONH_2_ production rate is achieved at 105.61 ± 4.86 mmol g_cat_^−1^ h^−1^ (Fig. 2a) from the co-oxidation of CH_3_CH_2_OH and NH_3_, which is a significant progression as it reaches the hundred-mmol level for photocatalytic C-N coupling under ambient conditions. Then, as the complete redox reaction proceeds in a heterogeneous photocatalysis process, it is deduced that the co-oxidation reaction is contributed by the light-generated h^+^ and O_2_-enabled active species, in which the detailed mechanism requires further investigation. Meanwhile, the hydrogen gas (H_2_) is yielded by the cooperative e^−^-driven reduction reaction with the dehydrogenated hydrogen from CH_3_CH_2_OH and NH_3_ (Supplementary Note 13 and Supplementary Figs. 10, 11), in which a minor amount of H_2_ is detected due to competing O_2_ activation and reduction for e^−^ consumption.

Subsequently, the CH_3_CONH_2_ selectivity is evaluated, corresponding to NH_3_ (N) and CH_3_CH_2_OH (C), respectively (Supplementary Note 15). As depicted in Fig. 2b and Supplementary Figs. 12–20, the concentration of CH_3_CONH_2_ gradually increases along with the consumption of NH_3_. It is noted that trace N_2_ byproduct is generated at the first 30 min, which originates from the NH_3_ peroxidation. However, as the reaction proceeds, the accumulation of N_2_ is impeded due to the decrease of the NH_3_ concentration, which illustrates that the controllable provision of the initial NH_3_ concentration is vital to determine the oxidative pathways of the system. The concentrations of the other byproducts are exceedingly low, resulting in data points in Fig. 2b that are so closely aligned they appear to overlap, making individual distinctions hard to discern. Under the limited provision of NH_3_ (100.00 mg L^−1^), a near-complete conversion of NH_3_ (97.67% ± 1.67%) for CH_3_CONH_2_ production is accomplished under the 60 mins’ light irradiation, presenting a superior CH_3_CONH_2_ selectivity (N) of 99.17 ± 0.39%. In addition, it is observed that the synthesis rate decreases after 45 min, which is reasonable due to the remaining NH_3_ concentration being low after rapid consumption. Based on these results, the recovery of the reaction rate and maintaining of selectivity are expected by conducting cycled experiments through periodical input of limited NH_3_.

Then, the CH_3_CONH_2_ selectivity regarding CH_3_CH_2_OH is also evaluated by comprehensively detecting the oxidative products in both the liquid and gaseous phases (Fig. 2c and Supplementary Figs. 21–25). The corresponding selectivity is ~100% at the first 30 min. Along with the consumption of NH_3_, the generation of the trace byproduct (CH_3_COOH) is observed due to the peroxidation of CH_3_CH_2_OH under low NH_3_ concentration, which again verifies the importance of limited NH_3_ provision to guarantee the superior selectivity. After the photocatalysis reaction for 1 h, the CH_3_CONH_2_ selectivity (C) is maintained at as high as 95.60 ± 0.22%. The reaction parameters of NH_3_ concentration for the CH_3_CONH_2_ selectivity test regarding NH_3_ and CH_3_CH_2_OH are set differentially. Specifically, a lower concentration of NH_3_ (100.00 mg L^−1^) is provided for its efficient conversion to evaluate the selectivity (N). While more NH_3_ (800.00 mg L^−1^) is included to impede the peroxidation of CH_3_CH_2_OH after the rapid NH_3_ consumption, where objective evaluations can be established for the selective oxidation of NH_3_ and CH_3_CH_2_OH for CH_3_CONH_2_ synthesis, respectively (Supplementary Note 17). These selectivity results imply that the key C- and N-intermediate must be clarified and optimized for the selective C-N coupling, which will, in turn, impede the individual peroxidation of CH_3_CH_2_OH and NH_3_. Moreover, when compared with the conventional industrial synthesis route (Supplementary Table 2), the photocatalytic co-oxidation route for CH_3_CONH_2_ synthesis demonstrates significant progress in terms of synthesis conditions, raw material price, and synthesis efficiency.

After systematically optimizing the reactants and reaction parameters, the establishment of the highly effective and selective co-oxidation route of CH_3_CH_2_OH and NH_3_ lays the theoretical foundation for developing the photocatalytic CH_3_CONH_2_ synthesis route. In comparison with the other reported routes for photocatalytic C-N coupling (Fig. 2d and Supplementary Table 3), it is significant that a high efficiency is accomplished by applying the photocatalytic co-oxidation routes, including the important indexes of synthesis rate of the targeted coupling products and the corresponding selectivity. Moreover, the as-constructed reaction system has been continuously operated with sufficient CH_3_CH_2_OH provision for 300 h (Supplementary Table 1), in which periodic NH_3_ conversion for stable CH_3_CONH_2_ production is maintained, delivering a gram scale yield of CH_3_CONH_2_ (1.82 g, Fig. 2e). The collected solid sample, with rotary evaporation for its recovery, is verified as CH_3_CONH_2_ by the XRD and nuclear magnetic resonance (^1^H NMR) technologies, consistent with the crystallography open database (COD #9007523, Fig. 2f) and chemical shift of the hydrogen atom (Supplementary Fig. 26) respectively. No deactivation of the P25 is observed based on the characterization results of the sample before and after the long-term stability test (Supplementary Figs. 27–30 and Supplementary Note 18).

### Reaction coordinates for the selective oxidation of CH_3_CH_2_OH and NH_3_

**Fig. 3:**
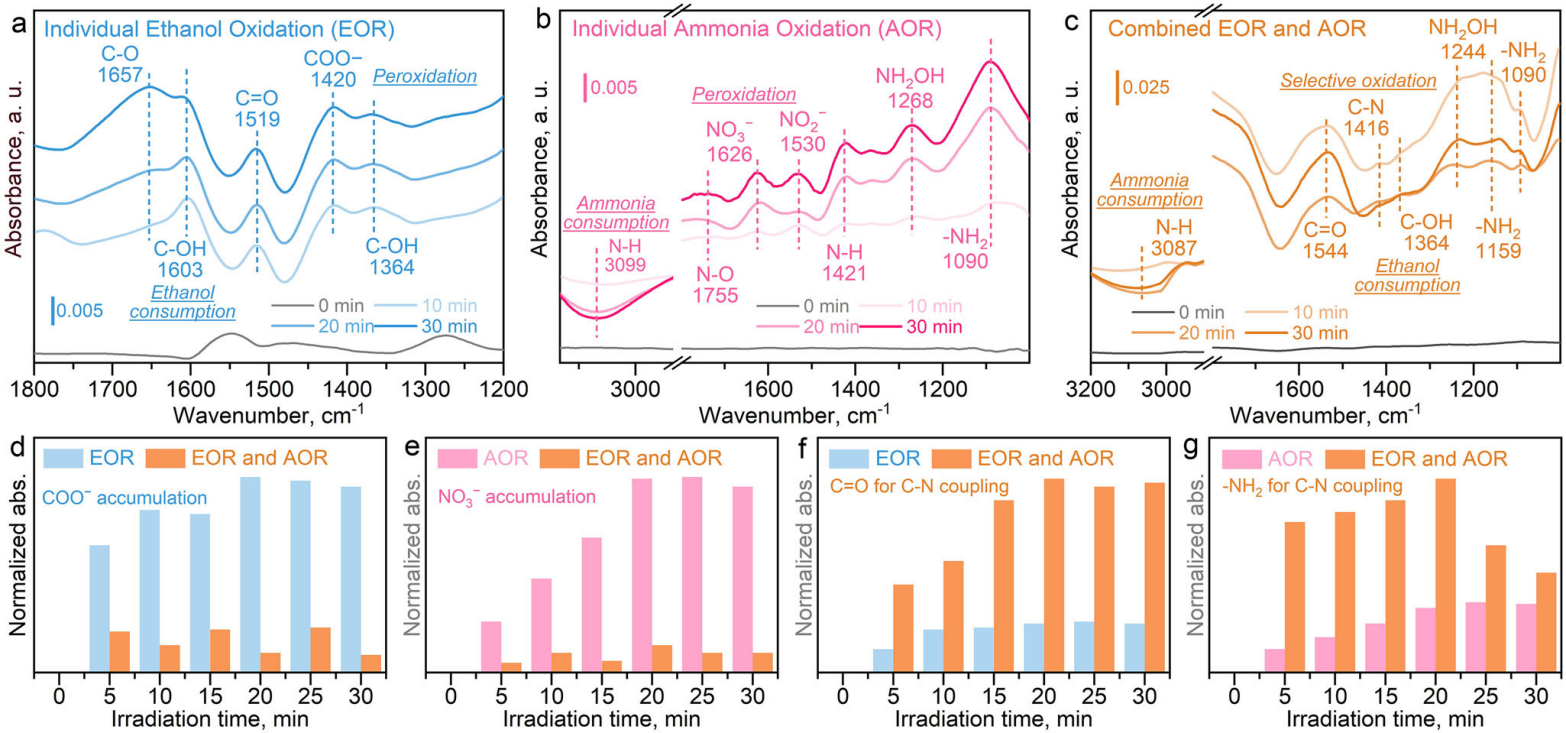
In situ ATR-FTIR investigation for revealing the reaction coordinates. Time-dependent IR signals for the individual ethanol oxidation reaction (EOR, **a**), ammonia oxidation reaction (AOR, **b**), and combined EOR and AOR (**c**), respectively; Normalized results of the IR signals of COO^−^ (**d**), NO_3_^−^ (**e**), C=O (**f**), and -NH_2_ (**g**), respectively. The full spectra of the adsorption and photocatalysis process are provided in Supplementary Information for individual EOR (Supplementary Fig. 37), individual AOR (Supplementary Fig. 38), and combined EOR and AOR (Supplementary Fig. 39), respectively.

To identify the critical step of the co-oxidation of CH_3_CH_2_OH and NH_3_. The variate-controlled in situ attenuated total reflection Fourier transform infrared spectroscopy (ATR-FTIR, Supplementary Fig. 36) investigation is conducted by comparing the IR signals for individual ethanol oxidation (EOR), ammonia oxidation (AOR) and their co-oxidation reactions. As the individual EOR proceeds (Fig. 3a, the spectra for the adsorption equilibrium process in the dark are displayed in Supplementary Fig. 37 and Supplementary Table 4), the gradual consumption of CH_3_CH_2_OH (C-OH at 1603 and 1364 cm^−1^) is observed^33^, which generates the corresponding oxidative intermediates (C-O at 1657 and C = O at 1519 cm^−1^)^34,35^. It is found that the peroxidative product (COO^−^ at 1420 cm^−1^)^36^ is produced by the excess oxidative ability in the individual EOR. Similarly, the consumption of NH_3_ (N-H at 3099 cm^−1^)^37^ yields the intermediated products of -NH_2_ (1090 cm^−1^)^38^ and NH_2_OH (1268 cm^−1^)^8^ in the individual AOR (Fig. 3b, the spectra for the adsorption equilibrium process in the dark are displayed in Supplementary Fig. 38 and Supplementary Table 5), in which the peroxidative products are also observed (N-O at 1755 cm^−1^, NO_2_^−^ at 1530 cm^−1^ and NO_3_^−^ at 1626 cm^−1^)^23,39^. Since NH_2_OH species are not detected during the reaction process (Fig. 2b, Supplementary Note 14, and Supplementary Fig. 20), it is clarified that NH_2_OH is not desorbed from the catalyst surface but is instead converted to other intermediates and products.

Most importantly, as illustrated in the combined co-oxidation reactions of EOR and AOR (Fig. 3c, the spectra for the adsorption equilibrium process in the dark are displayed in Supplementary Fig. 39 and Supplementary Table 6), as the co-oxidation of CH_3_CH_2_OH (C-OH at 1364 cm^−1^) and NH_3_ (N-H at 3087 cm^−1^) proceeds, the interaction of the C- (C=O at 1544 cm^−1^) and N-intermediates (-NH_2_ at 1159 and 1090 cm^−1^) leads to the generation of the coupling products as indicated by the C-N bond formation at 1416 cm^−1^ ^40^. Therefore, it is identified that the amide bond (-CONH_2_) can be effectively generated through the coupling of C=O and -NH_2_-based intermediates. However, their exact structures require further investigation.

Then, the signals for the peroxidative byproducts (Fig. 3d, e) and critical coupling intermediates (Fig. 3f, g) are normalized for comparison between the individual and combined oxidation reactions. The detailed methods for normalization of the FTIR signals are presented in Supplementary Note 8. It is observed that more peroxidative byproducts, including COO^−^ from EOR (Fig. 3d) and NO_3_^−^ from AOR (Fig. 3e), are accumulated in the respective individual oxidation reactions, which hinders the selective generation of key intermediates for C-N coupling. By the combination of EOR and AOR, the intensified signals for the intermediates of C=O (Fig. 3f) and -NH_2_ (Fig. 3g) are noted, leading to the directed coupling rather than the individual peroxidation. Furthermore, since the CH_3_CH_2_OH is sufficiently provided, the continuous accumulation of C=O species is reasonable, which provides sufficient C-intermediate for coupling. On the contrary, the limited provision of NH_3_ yields -NH_2_ at the first 20 min. Then -NH_2_ is consumed along with the decrease of NH_3_ concentration, which follows the efficiency test result (Fig. 2b). Hence, it is deduced that the -NH_2_-based species can be identified as the key N-intermediate which possesses higher reactive activity than that of the C=O species, due to its gradual accumulation and rapid consumption. It is again verified that continuous and effective C-N coupling can be accomplished by providing sufficient CH_3_CH_2_OH and periodically supplying limited NH_3_. This approach not only generates C- and N-intermediates for coupling but also avoids their peroxidation.

### Generation and coupling mechanism of the key intermediates

**Fig. 4:**
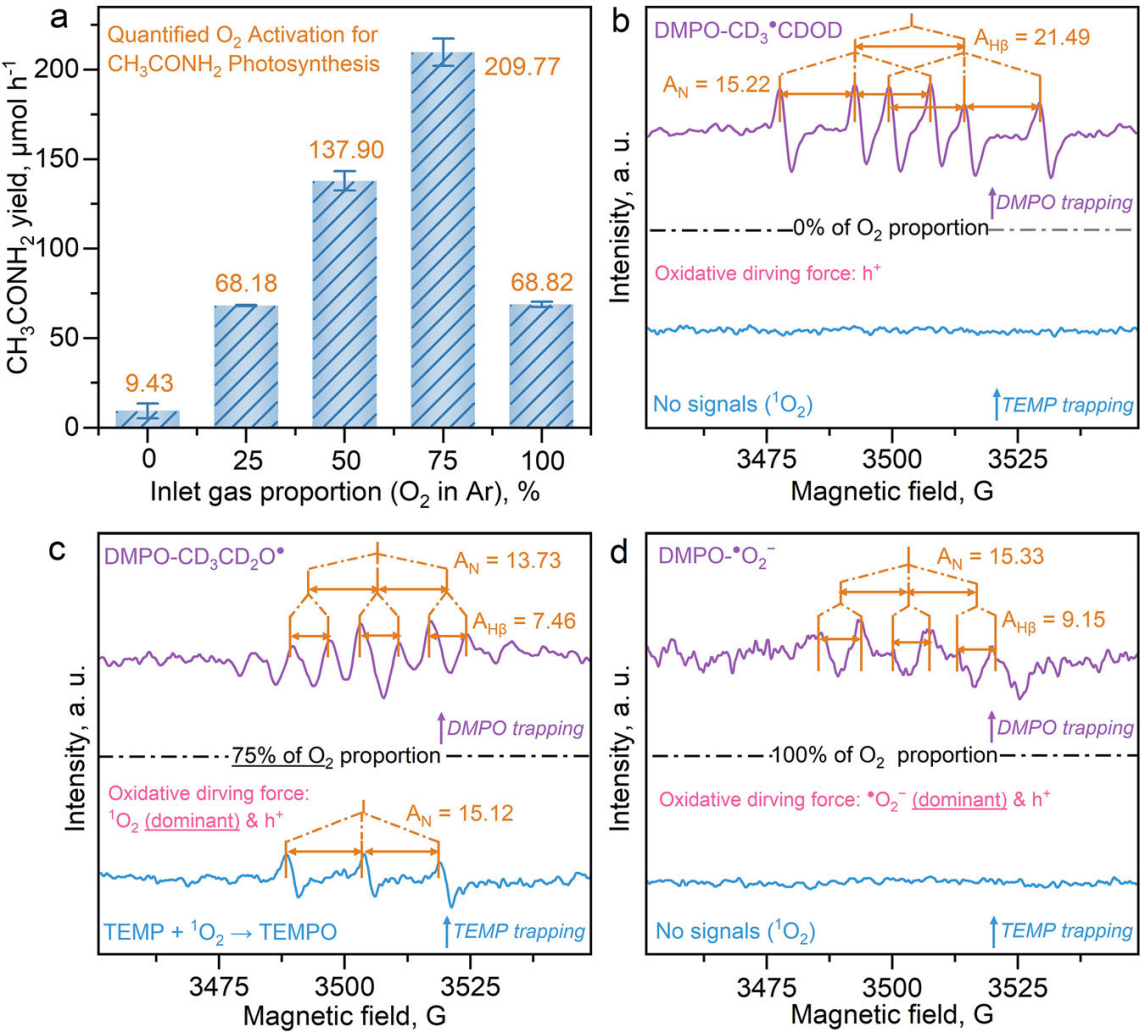
The mechanisms of O_2_ regulation for generating the coupling intermediates. **a** O_2_ proportion-dependent CH_3_CONH_2_ yield; DMPO-trapping (with D-labeling, images above) and TEMP-trapping (images below) in situ EPR experiments under the O_2_ proportion of 0% (**b**), 75% (**c**), and 100% (**d**) respectively. The error bars in (**a**) were drawn based on the calculated standard error of two parallel tests.

Based on the in situ ATR-FTIR investigation, it is acknowledged that the directed coupling of the C- and N-intermediates is vital to selectively producing CH_3_CONH_2_. Then, the generation pathways and coupling mechanism of these key intermediates should be revealed. The O_2_-proportion-dependent CH_3_CONH_2_ synthesis efficiency is first evaluated to identify the oxidative driving force for the generation of these intermediates (Fig. 4a and Supplementary Figs. 40,41). It is observed that the CH_3_CONH_2_ yield increases along with the elevation of O_2_ proportion (in Ar) from 0% to 75%, reaching a yield of 209.77 ± 7.68 μmol h^−1^. However, a rapid decrease of CH_3_CONH_2_ yield (68.82 ± 1.56 μmol h^−1^) is noted under excess O_2_ provision (100%), which directly confirms that the O_2_ molecules, as fundamental active species, require to be quantificationally provided for mild O_2_ activation, which enables the precise generation of the coupling intermediates. In addition, the production of e^−^-driven H_2_ is elevated at the O_2_ proportion of 0% than that of 75%, which again confirms that the O_2_ activation and reduction work as competing reactions with H_2_ evolution (Supplementary Fig. 42).

Then, a comprehensive iso-type (deuterium, D) labeled in situ electron paramagnetic resonance (EPR) experiment is conducted to dynamically track the selective EOR pathways and corresponding intermediates, by applying the 5,5-dimethyl-1-pyrroline *N*-oxide (DMPO) and 2,2,6,6-Tetramethylpiperidinooxy (TEMPO) as the respective trapping agents under different O_2_ proportion (Fig. 4b–d)^41–43^. The CD_3_CD_2_OD, rather than the other common reagents such as CH_3_OH or H_2_O, is applied as the only solvent for the D-labeled in situ EPR tests to precisely reproduce the selective EOR for acetamide synthesis. Under the 0% of O_2_ proportion, it is found that the EOR proceeds under the oxidative driving force of h^+^, as evidenced by the successful detection of the oxidative intermediate of the DMPO trapped alkyl radical (DMPO-CD_3_^●^CDOD) and the absence of the O_2_-enabled active species (Fig. 4b and Supplementary Figs. 43–45). Interestingly, DMPO trapped alkoxy radical (DMPO-CD_3_CD_2_O^●^) and trace DMPO-CD_3_^●^CDOD are both detected under the condition of an O_2_ proportion of 75%, which follows the optimum O_2_ proportion parameter for acetamide synthesis (Fig. 4c, Supplementary Fig. 46, 47, and Supplementary Note 19), illustrating the direct relationship between CD_3_CD_2_O^●^ generation and acetamide yield promotion. There is a tautomerization transformation from CD_3_^●^CDOD to CD_3_CD_2_O^●^, which is driven by the mild oxidative ability of singlet oxygen (^1^O_2_, Fig. 4c and Supplementary Fig. 48). The ^1^O_2_ is generated from either the activation of the triplet oxygen (^3^O_2_) or the h^+^-triggered rapid oxidation of superoxide radical (^●^O_2_^−^)^44,45^. Since ^3^O_2_ and ^●^O_2_^−^ are difficult to distinguish in the current in situ EPR experiments, it is reasonable to infer that both of these two reaction pathways are involved for the generation of ^1^O_2_. Moreover, the generation of CD_3_CD_2_O^●^, which is an O-centered active radical, implies that the C-OD bond in the CD_3_CD_2_OD molecule can be effectively activated by dehydrogenation, which yields the precursor of -C=O species for C-N coupling. As the concentration of O_2_ increases, the signal intensity of ^●^O_2_^−^ radical gradually increases while the signal intensity of ^1^O_2_ gradually decreases. Finally, the excess provision of O_2_ (100%) leads to the accumulation of the strong oxidative ^●^O_2_^−^ radicals (Fig. 4d, Supplementary Fig. 49, and Supplementary Note 20), in which no signals of the ^1^O_2_ are detected (Fig. 4d and Supplementary Figs. 50, 51). As the ^●^O_2_^−^ radicals bind to the reactants to generate peroxidative products, ^1^O_2_ species are no longer generated. Hence, the ^●^O_2_^−^-induced peroxidation is inevitable under such a high O_2_ concentration, resulting in the decreased coupling efficiency.

**Fig. 5:**
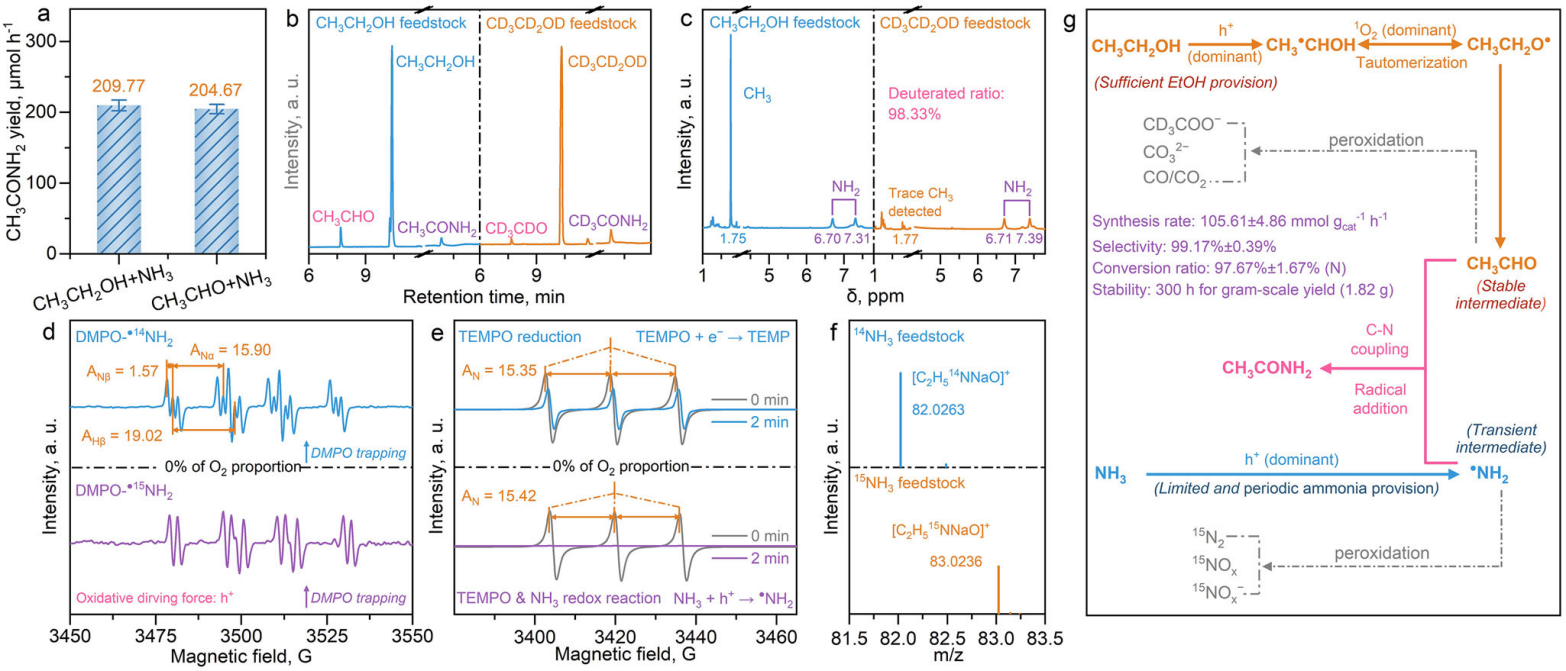
Transformation and coupling mechanism for the C- and N-intermediates. **a** Control experiments for CH_3_CONH_2_ synthesis by replacing CH_3_CH_2_OH with CH_3_CHO as the C-source; GC (**b**) and ^1^H NMR (**c**) results with D- (images right) and H-labeling (images left) respectively; **d** DMPO-trapping in situ EPR experiments with ^14^N- (image above) and ^15^N-labeling (image below) respectively; **e** in situ EPR spectra for e^−^-induced TEMPO consumption with (image below) and without (image above) NH_3_ provision respectively; **f** HR-MS results for CH_3_CONH_2_ detection with ^14^N- (image above) and ^15^N-labeling (image below) respectively; **g** Proposed reaction pathways of CH_3_CH_2_OH and NH_3_ co-oxidation for intermediates coupling and CH_3_CONH_2_ synthesis. The error bars in (**e**) were drawn based on the calculated standard error of two parallel tests.

By the quantified O_2_ activation at the proportion of 75%, it is confirmed that the peroxidation of CD_3_CD_2_O^●^ is avoided. Therefore, it is reasonably deduced that stable C-intermediate of acetaldehyde (CH_3_CHO), which possesses the critical -C=O group, can be generated through the hydrogen abstraction from CD_3_CD_2_O^●^. The corresponding control experiment is thereby conducted by replacing CH_3_CH_2_OH with CH_3_CHO as the initial reactant for C-N coupling (Fig. 5a, Supplementary Note 10, Supplementary Fig. 52, and Supplementary Table 7). It is noted that almost complete recovery of the CH_3_CONH_2_ yield is accomplished via the NH_3_-CH_3_CHO condensation (204.67 ± 6.58 μmol h^−1^), consistent with that of the co-oxidation of NH_3_ and CH_3_CH_2_OH (209.77 ± 7.68 μmol h^−1^), which identifies the CH_3_CHO as the stable C-intermediate, where the generation of CH_3_CHO requires precise regulation to avoid the decrease of the acetamide. Furthermore, the key role of CH_3_CHO is illustrated by the gas chromatography (GC, Fig. 5b) and corresponding mass spectra (MS, Supplementary Figs. 53, 54 and Supplementary Note 21) detection results. The provision of CH_3_CH_2_OH/CD_3_CD_2_OD feedstock results in the generation of H/D labeled acetaldehyde and acetamide, respectively, presenting a selective EOR along the route of CH_3_CH_2_OH → CH_3_^●^CHOH ↔ CH_3_CH_2_O^●^ → CH_3_CHO → CH_3_CONH_2_. Also, it is clarified by the ^1^H NMR (Fig. 5c, Supplementary Figs. 55 and 56 and Supplementary Note 22) results that the CD_3_CONH_2_ is detected with a deuterated ratio of 98.33%, which verifies the reliability and practicability of these designed D-labeled in situ experiments.

After successfully identifying the EOR for accumulating the stable C-intermediate of CH_3_CHO, the cooperative AOR mechanism is revealed by a series of ^15^N-/^14^N-labeled tracking experiments. The DMPO-^●14^NH_2_ and DMPO-^●15^NH_2_ are detected without O_2_ provision, respectively (Fig. 5d, Supplementary Figs. 57, 58, and Supplementary Note 23)^46,47^, manifesting that h^+^ is the dominant driving force for the mild oxidation of NH_3_ for ^●^NH_2_ generation. By combining the in situ ATR FTIR and in situ EPR spectra (Fig. 3c, g, 4h), it is indicated that the absorbed -NH_2_ is oxidized by h^+^ to produce ^●^NH_2_, which is involved in subsequent C-N coupling processes. Besides, further in situ EPR experiments are applied by using TEMPO as the indicator. Under the condition of individual TEMPO provision (Fig. 5e and Supplementary Fig. 59), a slight decrease of TEMPO signals is recorded due to the TEMPO reduction driven by e^−^ under light irradiation for 2 min On the contrary, near-complete TEMPO consumption is observed when adding NH_3_ into the test solution (Fig. 5e and Supplementary Fig. 60). Since no other oxidative driving force is included due to the absence of O_2_ (proportion at 0%), it is again confirmed that the selective AOR is dominantly driven by h^+^ for ^●^NH_2_ generation, which consumes more h^+^ and in turn provides extra e^−^ for accelerating TEMPO reduction.

By the combination of the efficiency test (Figs. 2b, 4a), the in situ ATR-FTIR results (Fig. 3c), as well as the ^15^N-labeled in situ EPR investigations for ^●^NH_2_ detection (Fig. 4h, i), it is acquired that no peroxidative N-containing intermediates or products are generated in the combined EOR and AOR under quantified O_2_ activation, which leads to the ~100% selectivity (N) for CH_3_CONH_2_ synthesis. Hence, it is concluded that the ^●^NH_2_ is identified as the transient N-intermediate for C-N coupling. Since the reaction rate coefficient of NH_3_-to-NH_2_ oxidation is an order of magnitude faster than that of the NH_2_-to-NH oxidation, the selective oxidation of NH_3_ primarily yields NH_2_-based intermediates or radicals than that of the NH^48,49^. Moreover, the fast formation (~1 ps) and relatively long lifetime (>1 ns) endow the ^●^NH_2_ radicals with a high possibility to participate in the coupling reactions, in comparison with the other N-centered radicals possessing short lifetimes, such as ^●^NH_4_ (13 ps)^50,51^, which again verifies that ^●^NH_2_ can act as the transient N-intermediate for C-N coupling. The illustration for ^●^NH_2_ detection parameters, especially the O_2_ proportion, is listed in Supplementary Note 24, which explains that the absence of the O_2_ provision is indispensable for the detection of ^●^NH_2_. Based on these results, it is clarified that the controllable generation and fast addition of transient ^●^NH_2_ radicals on stable CH_3_CHO intermediate should contribute directly to the efficient and selective C-N coupling for CH_3_CONH_2_ synthesis. The AOR pathways, along with the route of NH_3_ → ^●^NH_2_ → CH_3_CONH_2_, are then verified by the ^14^N/^15^N labeled high-resolution MS (HR-MS, Fig. 5f, Supplementary Figs. 61,62, and Supplementary Note 25) results, in which both CH_3_CO^14^NH_2_ and CH_3_CO^15^NH_2_ are detected, by introducing ^14^NH_3_ and ^15^NH_3_ (in CH_3_CH_2_OH) as the feedstocks respectively.

After these comprehensive mechanism investigations of the reaction pathways and coupling intermediates, the heterogeneous photocatalytic scheme for CH_3_CONH_2_ synthesis is proposed (Fig. 5g). Under the sufficient provision of CH_3_CH_2_OH and quantified O_2_ activation (75% in Ar), the CH_3_CHO, from selective oxidation of CH_3_CH_2_OH, is generated and accumulated as the stable C-intermediate for coupling. Meanwhile, the limited and periodic provision of NH_3_ leads to the controllable generation and fast addition of transient N-intermediate (^●^NH_2_) on stable CH_3_CHO intermediate, in which the rapid consumption of ^●^NH_2_ for C-N coupling avoids its peroxidation for side product generation. The coupling of long-lived C-intermediate and short-lived N-intermediate ensures a high selectivity and yield rate of CH_3_CONH_2_. The C-N coupling selectivity can be effectively maintained by the tailored oxidative ability and oxidative species, including h^+^ and e^−^-driven O_2_-enabled active species (^1^O_2_ and ^●^O_2_^−^), in which the excess e^−^ contributes to the H_2_ evolution to construct a complete photocatalytic redox reaction. For the overall photocatalytic synthesis pathway of CH_3_CONH_2_, optimizing the interaction between the initial reactive species and the photogenerated charge carriers is of crucial importance to achieve carrier-driven radical generation. By fully leveraging the radical characteristics of lifetime and migration distance, radical-mediated photocatalytic reactions can proceed effectively, which ensures the high efficiency of mass transfer and the sufficient utilization of light energy, thereby achieving a significant enhancement of the redox efficiency.

Here, we have demonstrated a compelling solar-driven photocatalytic synthesis system. By combining mild co-oxidation of CH_3_CH_2_OH and NH_3_, efficient and selective access to CH_3_CONH_2_ synthesis is achieved with heterogeneous photocatalysis technology. Through the precise regulation of the O_2_ activation and e^−^-h^+^ carriers, the CH_3_CONH_2_ yield reaches the gram scale with satisfying production rate, selectivity, and stability. Comprehensive mechanism investigation results indicate that the controllable generation and fast addition of the transient ^●^NH_2_ radical on stable CH_3_CHO intermediate contributes essentially to this catalytic performance in acetamide synthesis. The C-N coupling from stable and transient intermediates could provide scientific and technical feasibility for the solar light-driven synthetic fields, which, with further promotion, might be industrially and economically practical, thereby illuminating valuable opportunities for the mild-photocatalysis-driven synthetic industry.

## Methods

### Photocatalytic synthesis route and efficiency evaluation

Different N-source (NH_3_, KNO_2_, or KNO_3_) was added into 50.00 mL of absolute methanol (CH_3_OH), ethanol (CH_3_CH_2_OH), formic acid (HCOOH), or acetic acid (CH_3_OOH) to screen the C-N coupling routes (Supplementary Note 1). After the construction of the route of CH_3_CH_2_OH and NH_3_ co-oxidation for CH_3_CONH_2_ synthesis, the parameters of NH_3_ concentration, CH_3_CH_2_OH volume, O_2_ proportion, and catalyst dosage were optimized for elevating the synthesis efficiency (Supplementary Note 2). The corresponding parameters of the selectivity test regarding N and C were also optimized (Supplementary Notes 4,5). The reactants, intermediates, and products, in liquid and gaseous phases, were detected and quantified by high-performance liquid chromatography (HPLC, Shimadzu Essentia LC-16i/MSD), ion chromatography (IC, Shimadzu IC-16) and infrared flue gas analyzer (Bruker MATRIX-MG5), respectively (Supplementary Note 26).

After the long-term stability test, the collected catalyst sample was characterized by the X-ray diffraction technology (XRD, Shimadzu XRD-6100) for its crystal stability, scanning electron microscopy (SEM, FEG ESEM XL30) and transmission electron microscopy (TEM, FEI Talos F200S) for its geometric stability respectively. The obtained reaction mixture was centrifuged for the separation of the solution and catalyst. Then, a rotary evaporator was applied to remove the solvent and potential side products from the solution. The collected product sample was characterized by the XRD (Supplementary Notes 6, 7).

### In situ ATR-FTIR investigation

An INVENIO R FTIR (Bruker) spectrometer equipped with a mercury cadmium telluride (MCT) detector was utilized for the measurements. Before the test, 100.0 uL of catalyst ink (a mixture of 10.00 mg of P25, 25.0 uL of Nafion, and 1.00 mL of CH_3_CH_2_OH) was deposited onto the Si crystal and then dried in air. The reaction chamber was filled with a total of 10.00 mL of the reaction solution, and 75% of O_2_ (in Ar) was continuously injected into the system. An Xe lamp (Bobei BBZM-1) was applied as the light source, and detection was performed during light irradiation^52^.

The pristine IR signals were normalized to evaluate the species’ evolution directly. Between the columns of data, the highest value was set to be 1, and the lowest value was set to be 0. The rest were correspondingly normalized from 0 to 1. The resulting normalized data were thus described as a function of the IR scanning time^53^.

### In situ EPR investigation by H/D labeling

All the in situ EPR measurements were conducted on the equipment of Bruker EMX Nano. 1000.0 μL of CH_3_CH_2_OH (or CD_3_CD_2_OD), 40.0 μL of NH_3_·H_2_O, 50.0 μL of the well-mixed P25 suspension (1000.00 mg L^−1^ in CH_3_CH_2_OH or CD_3_CD_2_OD), 10.0 μL of DMPO were added into the reactor under the O_2_ proportion (in Ar) of 0, 75, and 100%, respectively. The EPR signals were recorded at 1, 3, 5, and 10 min under the dark or light irradiation conditions, respectively, collected by a capillary (Supplementary Notes 9).

### In situ EPR investigation by ^14^N/^15^N labeling

About 550.0 μL of ^14^NH_3_·H_2_O (10.00 g L^−1^) or ^15^NH_4_Cl (10.00 g L^−1^ of ^15^NH_4_^+^), 390.0 μL of DI and 50.0 μL of the well-mixed P25 suspension (in CH_3_CH_2_OH, 1000.00 mg L^−1^) were added into the reactor under the Ar (99.999%) gas injection. The EPR signals were recorded at 1, 3, 5, and 10 min under the dark or light irradiation condition respectively, collected by a capillary (Supplementary Notes 11, 12).

The other experimental details are provided in the Supplementary Information.

## Supplementary information

Supplementary Information

Peer Review File

## Source data

Source Data

## Data Availability

All source data generated in this study are provided in the Supplementary Information and Source Data files. Source data are provided with this paper.
