# Supplementary material for: Photocatalytic C-N coupling from stable and transient intermediates for gram-scale acetamide synthesis
Source: Nat Commun. 2025 Apr 15;16:3590. doi: 10.1038/s41467-025-58840-0 (PMC12000377; doi:10.1038/s41467-025-58840-0)
Supplement: Supplementary file 1 — Supplementary Information [file 41467_2025_58840_MOESM1_ESM.pdf]

Supplementary Information for

Photocatalytic C-N coupling from stable and transient intermediates for gram-scale acetamide synthesis

Xin Li[1], Weiping Yang[1], Junping Yue[2], Jieyuan Li[1*], Shujie Shen[1], Ruimin Chen[1], Jielin Wang[1], Huimin Dan[1], Dagang Yu[2] and Fan Dong[1*]

1 Research Center for Carbon-Neutral Environmental & Energy Technology, Institute of Fundamental and Frontier Sciences, University of Electronic Science and Technology of China, Chengdu 611731, China.

2 Key Laboratory of Green Chemistry & Technology of Ministry of Education, College of Chemistry, Sichuan University, Chengdu 610064, China.

* Corresponding authors:

Fan Dong (dongfan@uestc.edu.cn); Jieyuan Li (jieyli@uestc.edu.cn)

Table of contents

Supplementary Notes 1-26

Supplementary Figures 1-62

Supplementary Tables 1-7

Supplementary References 1-19

Supplementary Note 1 | Reaction route screening experiment.

300.00 mg L^{-1} of different N-source (NH_3, KNO_2 or KNO_3) and 35 mmol different C-source (CH_3OH, CH_3CH_2OH, HCOOH, CH_3OOH, CH_3COONa) were added into 50.00 mL acetonitrile, in which arranged 13 routes (Table 1 in the Manuscript). The 300.00 mg L^{-1} NH_3 is 0.5 vol.% of NH_3 gas (50 mL min^{-1}) continuously injected into the reactor. The 300.00 mg L^{-1} NO_2^- is 1.50 mL of NO_2^- (100000.00 mg L-1) dissolved in 50.00 mL of reaction solution. The 300.00 mg L^{-1} NO_3^- is 0.15 mL of NO_3^- (10000.00 mg L-1) dissolved in 50.00 mL of reaction solution. A quart photocatalysis reactor (MC-GF250, Merry Change, China) was applied with the introduction of 50.00 mg of P25 (Degussa Co. Ltd.) photocatalyst. The reaction temperature was set to 25℃ by circulating water and consistent stirring was set to 200 rpm. O_2 (99.999%, 30.0 mL min^{-1}) and Ar (99.999%, 10.0 mL min^{-1}) were continuously injected into the reaction solution. After the absorption-dissolution equilibrium was reached for 10 mins. A 300W Xe lamp (Merry Change MC-X301B) was turned on for the photocatalysis reaction. The liquid samples were extracted per 15 mins for product detection by high-performance liquid chromatography (HPLC, Shimadzu Essentia LC-16i/MSD), including the possible products of formamide ($HCONH_2$), acetamide (CH_3CONH_2) and urea [$CO(NH_2)_2$].

The products were analyzed by HPLC equipped with a photo-diode array (PDA) detector and a GIST C_{18}, 5 μm, 4.6 × 250 mm column. The corresponding mobile phase, flow rate, and detected wavelength for formamide were acetonitrile-water (10:90), 0.25 mL min^{-1}, and 198 nm. The corresponding mobile phase, flow rate, and detected wavelength for acetamide were acetonitrile-water (80:20), 0.5 mL min^{-1}, and 198 nm. The corresponding mobile phase, flow rate, and detected wavelength for urea were acetonitrile-water (10:90), 0.2 mL min^{-1}, and 198 nm.

Supplementary Note 2 | Parameters for the CH_3CONH_2 production rate.

1) Screening of the optimum NH_3 concentration: The total reaction volume was set to 50.00 mL, which consists of 49.00 mL of acetonitrile (CH_3CN) as the solvent and 1.00 mL of CH_3CH_2OH as the C-source. 100.00, 300.00, 500.00, 800.00 and 1000.00 mg L^{-1} of NH_3 (in CH_3CH_2OH) were added as the N-source, respectively. The other experimental details are the same as that of the Supplementary Note 1.

2) Screening of the optimum volume of CH_3CH_2OH: The total reaction volume was set to 50.00 mL using CH_3CN as the solvent, in which 1 vol.%, 2 vol.%, 4 vol.%, 10 vol.% and 100 vol.% of CH_3CH_2OH were injected as the C-source. 800.00 mg L^{-1} of NH_3 (in CH_3CH_2OH) was added as the N-source. The other experimental details are the same as that of the Supplementary Note 1.

3) Screening of the optimum O_2 proportion: The total reaction volume was set to 50.00 mL, which consists of 49.00 mL of CH_3CN as the solvent. 1.00 mL of CH_3CH_2OH and 800.00 mg L^{-1} of NH_3 (in CH_3CH_2OH) are introduced as the C- and N-sources respectively. 0%, 25%, 50%, 75% and 100% of the O_2 proportion (in Ar) were continuously injected as the feeding gas respectively. The other experimental details are the same as that of the Supplementary Note 1.

4) Screening of the optimum unit production rate per catalyst dosage: The total reaction volume was set to 50.00 mL, which consists of 49.00 mL of CH_3CN as the solvent. 1.00 mL of CH_3CH_2OH and 800.00 mg L^{-1} of NH_3 (in CH_3CH_2OH) are introduced as the C- and N-sources respectively. 75% of O_2 (in Ar) was continuously injected. 50.00 mg, 25.00 mg, 10.00 mg, 5.00 mg and 1.00 mg of P25 were dispersed in the reaction solution as the catalyst respectively. The other experimental details are the same as that of the Supplementary Note 1.

Supplementary Note 3 | Photocatalyst screening experiment.

1) The preparation of photocatalyst:

Commercial TiO_2 was purchased from Degussa Co. Ltd.

Anatase TiO_2 was purchased from Macklin Co. Ltd.

Nanosheets TiO_2 were prepared by the hydrothermal method by mixing 20.00 mL of tetrabutyl titanate and 3.00 mL of hydrofluoric acid solution in a dry Teflon-lined stainless-steel autoclave of 100.00 mL capacity and then stored in an electric furnace at 180 °C for 24h. After the hydrothermal reaction, the autoclave was cooled naturally to room temperature, and the white solid was separated by high-speed centrifugation. It was washed several times with ethanol and deionized water, dried at 80 °C for 12 h, and labeled as the TNS.

BiOCl: 4.00 mmol $Bi(NO_3)\cdot 5H_2O$ and 4.00 mmol KCl were dissolved in 100.00 mL deionized water, followed by vigorous stirring at room temperature for one hour. After the reaction, the precipitates were collected by centrifugation, washed with deionized water and ethanol, and dried at 60 °C in a vacuum. The obtained products were labeled as BiOCl.

BiOBr: 4.00 mmol $Bi(NO_3)\cdot 5H_2O$ and 4.00 mmol KBr were dissolved in 100.00 mL deionized water, followed by vigorous stirring at room temperature for one hour. The rest of the operation is consistent with BiOCl. The obtained products were labeled as BiOBr.

$Bi_2O_2CO_3$: 2.00 mmol $Bi(NO_3)\cdot 5H_2O$ were dissolved in 100.00 mL deionized water, and stirred at room temperature for one hour. Subsequently, 6.00 mmol urea was added to the solution and stirred for 30 min. The precipitates were separated by centrifugation and washed with deionized water and absolute ethanol three times. The obtained solids were collected and dried overnight at 180 °C, being further labeled as $Bi_2O_2CO_3$.

$Sr_2Sb_2O_7$: 5.00 mmol $C_4H_6O_4Sr$ and 2.50 mmol $K_2H_2Sb_2O_7$ were dissolved in 30.00 mL of deionized water. Under vigorous stirring, HNO_3 or NaOH was added dropwise into the solution until pH = 13. Then, the solution was moved into a 45.00 mL Teflon-lined autoclave and heated at 180 °C for 48 h. After cooling in the air, the obtained

mixture was centrifuged several times with deionized water and ethanol successively. Finally, the collected samples were dried in an oven at 60 °C for a whole night. The resulting product was labeled as $Sr_2Sb_2O_7$.

$g\text{-}C_3N_4$: 10.00 g of urea were placed in an alumina crucible with a cover. The crucible was heated to 550 °C at a heating rate of 15 °C·min^{-1} in a muffle furnace and maintained for 2 h. After the thermal treatment, the crucible was cooled down to room temperature in the muffle furnace. The samples collected were labeled as $g\text{-}C_3N_4$.

2) Photocatalyst screening experiment.

The total reaction volume was set to 50.00 mL, which consists of 49.00 mL of CH_3CN as the solvent. 1.00 mL of CH_3CH_2OH and 800.00 mg L^{-1} of NH_3 (in CH_3CH_2OH) are introduced as the C- and N-sources respectively. 75% of O_2 (in Ar) was continuously injected. 5.00 mg of different photocatalysts (such as commercial TiO_2, nanosheet TiO_2, anatase TiO_2, BiOCl, BiOBr, $Bi_2O_2CO_3$, $Sr_2Sb_2O_7$, and $g\text{-}C_3N_4$) were dispersed in the reaction solution respectively. The other experimental details are the same as that of the Supplementary Note 1.

Supplementary Note 4 | Details for the CH_3CONH_2 selectivity (N) test.

The total reaction volume was set to 50.00 mL, which consists of 49.50 mL of CH_3CN as the solvent. 0.50 mL of CH_3CH_2OH and 100.00 mg L^{-1} of NH_3 (in CH_3CH_2OH) are introduced as the C- and N-sources respectively. 75% of O_2 (in Ar) was continuously injected. 50.00 mg of P25 was applied as the catalyst. An ion chromatography (IC, Shimadzu IC-16) was used to detect the liquid products in the reaction solution, including NO_3^- (C_1) and NO_2^- (C_2). An Infrared flue gas analyzer (Bruker MATRIX-MG5) was applied to test the gaseous products in the tail gas, including N_2 (C_3), N_2O (C_4), NO (C_5) and NO_2 (C_6). The other experimental details are the same as that of the Supplementary Note 1. The CH_3CONH_2 selectivity for N-source (S_N) was calculated as follows:

$$S_N = \frac{C_{acetamide} * 100\%}{C_{acetamide} + C_1 + C_2 + C_3 + C_4 + C_5 + C_6}$$

Supplementary Note 5 | Details for the CH_3CONH_2 selectivity (C) test.

The total reaction volume was set to 50.00 mL, which consists of 49.00 mL of CH_3CN as the solvent. 1.00 mL of CH_3CH_2OH and 800.00 mg L^{-1} of NH_3 (in CH_3CH_2OH) are introduced as the C- and N-sources respectively. 75% of O_2 (in Ar) was continuously injected. 50.00 mg of P25 was applied as the catalyst. An IC (Shimadzu IC-16) was used to detect the liquid product of CH_3COO^- (C_1) in the reaction solution. An infrared flue gas analyzer (Bruker MATRIX-MG5) was applied to test the gaseous products in the tail gas, including CH_3CHO (C_2), CO (C_3) and CO_2 (C_4). The other experimental details are the same as that of the Supplementary Note 1. The CH_3CONH_2 selectivity for C-source (S_N) was calculated as follows:

$$S_C = \frac{C_{acetamide} * 100\%}{C_{acetamide} + C_1 + C_2 + C_3 + C_4}$$

Supplementary Note 6 | Details for the long-term stability test.

The total reaction consists of 50.00 mL of CH_3CH_2OH and 800.00 mg L^{-1} of NH_3 (in CH_3CH_2OH) are introduced as the C- and N-sources respectively. 75% of O_2 (in Ar) was continuously injected. 50.00 mg of P25 was applied as the catalyst. The NH_3 will be a periodic provision. At intervals of approximately 20 hours, when the concentration of NH_3 approaches depletion (falling below 100. 00 mg L^{-1}), an appropriate amount of NH_3 (35.00 - 40.00 mg) is added to the reaction system to restore the NH_3 concentration to a level of 800.00 mg L^{-1}. NH_3 was added 15 times in total within 0-300 hours, and the total amount of NH_3 added was 560.00 mg. The reaction solution was extracted for the CH_3CONH_2 test every 10 h, in which the catalyst was washed and dried for further testing and characterization. The collected catalyst samples were characterized by X-ray diffraction technology (XRD, Shimadzu XRD-6100) for its crystal stability, scanning electron microscopy (SEM, FEG ESEM XL30) and transmission electron microscopy (TEM, FEI Talos F200S) for its geometric stability respectively.

Supplementary Table 1 | Details for NH_3 periodic provision in the long-term stability test.

Reaction time (h)	Remaining concentration of NH_3 before periodic addition (mg L^{-1})	NH_3 periodic addition (mg)	Concentration of NH_3 after periodic addition (mg L^{-1})	Acetamide yield (g)
0	800.00	40.00	800.00	0
10	405.76	0	/	0.05891
20	82.62	36.00	800.07	0.11939
30	413.41	0	/	0.18289
40	90.55	36.00	809.21	0.23697
50	409.57	0	/	0.28967
60	64.77	38.00	801.06	0.36284
70	418.11	0	/	0.41532
80	67.73	37.00	805.17	0.48465
90	399.32	0	/	0.55299
100	70.81	38.00	812.65	0.60517
110	354.51	0	/	0.68499
120	47.85	39.00	819.74	0.73785
130	400.61	0	/	0.80223
140	71.57	36.00	798.44	0.85646
150	360.56	0	/	0.92579
160	49.02	38.00	797.95	0.98056
170	458.09	0	/	1.03091
180	95.55	35.00	805.01	1.08279
190	370.43	0	/	1.16159
200	74.97	40.00	804.42	1.21448
210	400.42	0	/	1.25391
220	90.17	35.00	800.75	1.31948
230	371.71	0	/	1.38358

240	86.38	36.00	806.38	1.43569
250	435.82	0	/	1.49498
260	105.42	36.00	825.38	1.54768
270	407.00	0	/	1.61673
280	76.21	40.00	796.35	1.69833
290	467.34	0	/	1.75066
300	72.29	0	/	1.81639
Note: NH_3 was added every 20 h, with the amount of 35.00-40.00 mg per addition (to control the NH_3 concentration at 800.00 mg L^{-1}). NH_3 was added 15 times in total within 300 h, and the total amount of NH_3 added was 560.00 mg.				

Supplementary Note 7 | Product separation and collection after long-term test.

The reaction mixture after the long-term stability test was centrifuged for the separation of solution and catalyst. Then rotary evaporator was applied to remove the solvent and potential side products from the solution at 43°C with a rotation speed of 60 rpm. The obtained solid sample was weighed and characterized by XRD.

Supplementary Note 8 | *In-situ* ATR-FTIR tests

For the *in-situ* ATR-FTIR test, an INVENIO R FT-IR (Bruker) spectrometer equipped with a mercury cadmium telluride (MCT) detector was utilized. All spectra were presented by the absorbance (-log (R/R_0)). The spectral resolution was 4 cm^{-1}, and each curve was scanned for 0.5 min. Before the test, 100.0 uL of catalyst ink (a mixture of 10.00 mg of catalyst, 25.0 uL of Nafion, and 1.00 mL of CH_3CH_2OH) was deposited onto the Si crystal and then dried in air. The reaction chamber was filled with a total of 10.00 mL of the reaction solution, and 75% of O_2 (in Ar) was continuously injected into the system. An Xe lamp (Bobei BBZM-1) was applied as the light source, and detection was performed during light irradiation.

The pristine IR signals were normalized to evaluate the species' evolution directly. For the specific species in the AOR process, EOR process, and AOR-EOR co-oxidation process, the data of peak heights were respectively extracted for all processes. Between the columns of data, the highest value was set to be 1 and the lowest value was set to be 0. The rest were correspondingly normalized from 0 to 1. The resulting normalized data were thus described as a function of the IR scanning time.

For instance, for the COO^- species detected at 1420 cm^{-1} in the EOR and EOR+AOR, the peak intensities at 1420 cm^{-1} were extracted from the spectra collected during the 0-30 min interval. The highest peak intensity within this range was normalized to 1, the lowest was set to 0, and the remaining values were linearly scaled between 0 and 1. The same normalization process was applied to the NO_3^- species in the AOR and EOR+AOR, represented by the peak at 1626 cm^{-1}, using the data from the 0-30 min interval. The procedure was also followed for the C=O species at 1544 cm^{-1} in the EOR and EOR+AOR, and the -NH_2 species at 1159 cm^{-1} in the AOR and EOR+AOR, where the maximum intensity within the 0-30 min range was set to 1, the minimum to 0, and all other values normalized accordingly. The normalized data obtained from this process are presented below:

Time (min)	COO^-		NO_3^-		C=O		$-NH_2$	
	EOR	EOR and AOR	AOR	EOR and AOR	EOR	EOR and AOR	EOR	EOR and AOR
0	0	0	0	0	0	0	0	0
5	0.65	0.21	0.26	0.05	0.12	0.45	0.12	0.77
10	0.83	0.14	0.48	0.10	0.22	0.57	0.18	0.82
15	0.81	0.22	0.69	0.06	0.23	0.88	0.25	0.88
20	1.00	0.10	0.99	0.14	0.25	1.00	0.33	1.00
25	0.98	0.23	1.00	0.10	0.26	0.95	0.36	0.65
30	0.95	0.09	0.95	0.10	0.25	0.97	0.35	0.51

Supplementary Note 9 | *In-situ* EPR tests by H/D iso-type labeling.

All the *in-situ* EPR measurements were conducted on the equipment of Bruker EMX Nano.

1) O_2 proportion-dependent DMPO-trapping experiments: 1000.0 μL of CH_3CH_2OH (or CD_3CD_2OD), 40.0 μL of $NH_3 \cdot H_2O$, 50.0 μL of the well-mixed P25 suspension (1000.00 mg L^{-1} in CH_3CH_2OH or CD_3CD_2OD), 10.0 μL of DMPO were added into the reactor under the O_2 proportion (in Ar) of 0%, 75% and 100% respectively. The EPR signals were recorded at 1, 3, 5 and 10 mins under the dark or light irradiation condition respectively, collected by a capillary.

2) NH_3 concentration-dependent TEMP-trapping experiment: 1000.0 μL of CH_3CH_2OH, 40.0 μL of $NH_3 \cdot H_2O$, 50.0 μL of the well-mixed P25 suspension (1000.00 mg L^{-1} in CH_3CH_2OH or CD_3CD_2OD), 50.0 μL of TEMPO were added in the reactor under the O_2 proportion (in Ar) of 0%, 75% and 100% respectively. The EPR signals were recorded at 1, 3, 5 and 10 mins under the dark or light irradiation condition respectively, collected by a capillary. The generation of TEMPO from the oxidation of TEMPO by $^1O_2$ was recorded, which clarified the generation and accumulation of $^1O_2$.

Supplementary Note 10 | Replacing the CH_3CH_2OH with CH_3CHO as the C-source for efficiency evaluation.

50.00 mg of P25 was dispersed in 49.00 mL of the CH_3CN solvent. 0.5 vol.% of NH_3 gas (50 mL min^{-1}) was continuously injected into the reactor. 1.00 mL of CH_3CH_2OH or CH_3CHO was added when the NH_4^+ concentration reached 300.00 mg L^{-1}. Then the injection of NH_3 gas was adjusted to O_2 (75% in Ar). The other experimental details are the same as that of the Supplementary Note 1.

Supplementary Note 11 | Detection of H/D labeled C-species and $^{14}N/^{15}N$ labeled N-source.

1) Detection of the H/D labeled C-species: 50.00 mg of P25 was dispersed in 49.00 mL of the CH_3CN solvent. 0.5 vol.% of NH_3 gas (50 mL min^{-1}) was continuously injected into the reactor. 1.00 mL of CH_3CH_2OH or CD_3CD_2OH was added when the NH_4^+ concentration reached 300.00 mg L^{-1}. Then the injection of NH_3 gas was adjusted to O_2 (75%). The collected solid sample was tested by the GC-MS (Agilent 7890B GC system, Agilent 5677A mass spectrometer). After the photocatalysis reaction, the supernate was collected by centrifugation and rotary evaporation, which was then tested by the ^1H NMR (Bruker Advance 400) after dissolution in D-labeled DMSO solvent. The reaction procedure of the rotary evaporation was the same as that of Supplementary Note 6.

2) Detection of the $^{14}N/^{15}N$ labeled N-species: 50.00 mg of P25 was dispersed in 49.00 mL of the CH_3CH_2OH solvent. 300.0 μL of $^{14}NH_4^+/^{15}NH_4^+$ (10.00 g L^{-1}, $^{14}NH_4Cl/^{15}NH_4Cl$) solution was added respectively. 5% of the O_2 proportion (in Ar) was continuously injected as the feeding gas. After the photocatalysis reaction for 2 h, the supernate was collected by centrifugation and rotary evaporation, which was then tested by the HR-MS (SHIMADZU LCMS-IT-TOF). The reaction procedure of the rotary evaporation was the same as that of Supplementary Note 6.

Supplementary Note 12 | *In-situ* EPR tests by $^{14}N/^{15}N$ iso-type labeling

1) Detection of the $^{\bullet 14}NH_2$ and $^{\bullet 15}NH_2$: 550.0 μL of $NH_3 \cdot H_2O$ (10.00 g L^{-1}) or $^{15}NH_4Cl$ (10.00 g L^{-1} of $^{15}NH_4^+$), 390.0 μL of DI, 50.0 μL of the well-mixed P25 suspension (in CH_3CH_2OH, 1000.00 mg L^{-1}) and 10.0 μL of DMPO were added into the reactor under the Ar (99.999%) gas injection. The EPR signals were recorded at 1, 3, 5 and 10 mins under the dark or light irradiation condition respectively, collected by a capillary.

2) Detection of TEMPO signals: 2000.0 μL of CH_3CH_2OH, 50.0 μL of $NH_3 \cdot H_2O$ (10.00 g L^{-1}), 50.0 μL of the well-mixed P25 suspension (in CH_3CH_2OH, 1000.00 mg L^{-1}) and 10.0 μL of TEMPO (50.00 g L^{-1}) were added into the reactor under the continuous Ar (99.999%) gas injection. The EPR signals were recorded at 1, 2, 3 and 4 mins under the dark or light irradiation condition respectively, collected by a capillary. The generation and transformation of light-excited e^- were recorded by the reduction of TEMPO with and without NH_3 respectively, which in turn clarified the generation h^+ and its interaction with NH_3.

Supplementary Note 13 | Experimental details for H_2 detection from the e^--driven reduction reactions

The total reaction volume was set to 50.00 mL, which consists of 50.00 mL CH_3CH_2OH and 800.00 mg L^{-1} of NH_3 (in CH_3CH_2OH). 0% and 75% of the O_2 proportion (in Ar) were continuously injected as the feeding gas respectively. The other experimental details are the same as that of the Supplementary Note 1. The gas samples were extracted per 15 mins for H_2 detection by a GC-TCD (CEAULIGHT GC-7920).

Supplementary Note 14 | Experimental details for NH_2OH detection through colorimetric method.

Firstly, 1.00 mL of the reaction solution with the photocatalyst filtered out was diluted with 1.00 mL of 0.05 M phosphate buffer solution (pH=6.8) and 0.80 mL of deionized water. Then, 0.20 mL of trichloroacetic acid, 1.00 mL of 1% 8-quinolinol (w/v ethanol), and 1.00 mL of 1 M Na_2CO_3 solution were injected into the above solution. Finally, the solution was heated in a water bath under the dark at 100 °C for 1 min, and then the absorbance at 709 nm was measured using a UV-visible absorption spectrophotometer.

Supplementary Note 15 | The reasonableness of multiple methods for detecting C/N species.

For the Efficiency evaluation for acetamide photosynthesis (unit production rate, C/N selectivity), we chose to evaluate the activity of C-N coupling: using the combination of multiple detection techniques:

Gas-phase products: N species including N_2, N_2O, NO, NO_2 and C species including CO_2, CO and CH_3CHO are detected by an infrared flue gas analyzer.

Liquid phase products: NH_4^+ by cation chromatography, NO_3^-, NO_2^- and $CH3COO^-$ by anion chromatography and the target product CH_3CONH_2 by HPLC.

Through the above methods, we can comprehensively test all possible C/N products and achieve scientific performance evaluation.

All source data in Supporting Imformation are provided in the “Source data for Supplementary” Files.

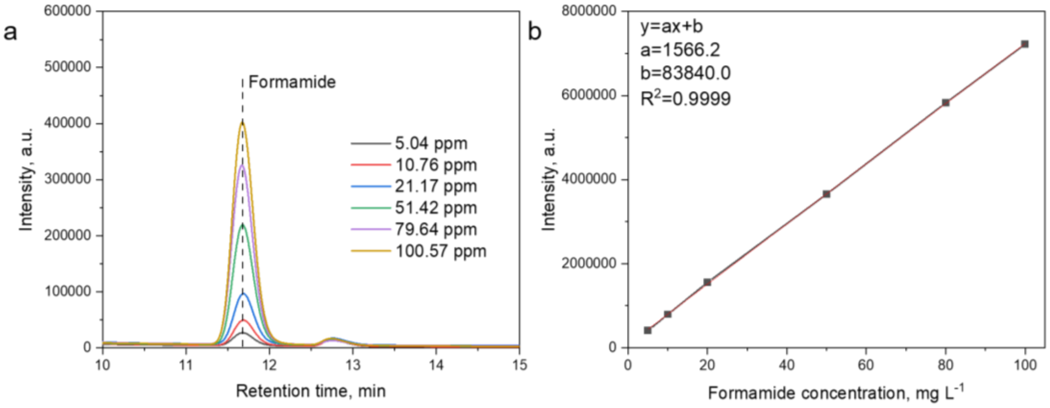

Supplementary Figure 1 | Standard curves. Tested standard intensities (a) and standard curves (b) for formamide ($HCONH_2$) detection by HPLC.

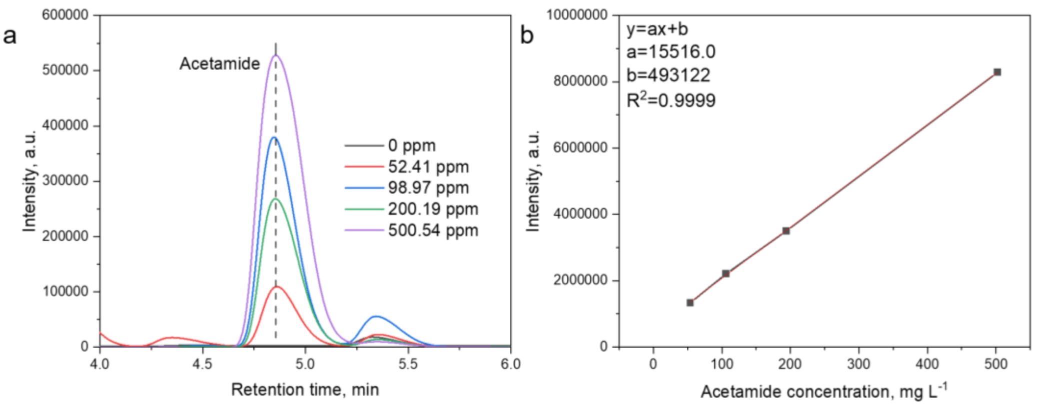

Supplementary Figure 2 | Standard curves. Tested standard intensities (a) and standard curves (b) for acetamide (CH_3CONH_2) detection by HPLC.

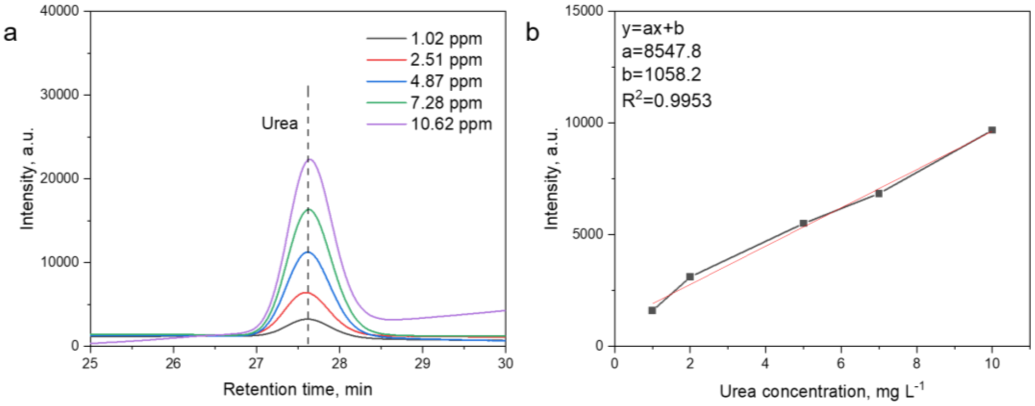

Supplementary Figure 3 | Standard curves. Tested standard intensities (a) and standard curves (b) for urea [$CO(NH_2)_2$] detection by HPLC.

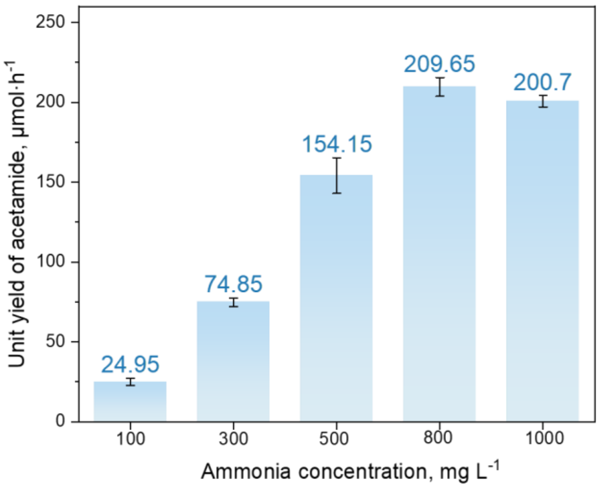

Supplementary Figure 4 | Screening experiments. Screening of the optimum NH_3 concentration for acetamide synthesis. The error bars were drawn based on two parallel experiments.

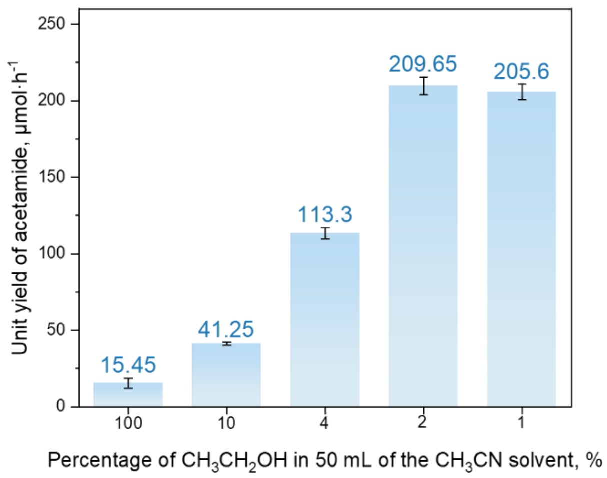

Supplementary Figure 5 | Screening experiments. Screening of the optimum CH_3CH_2OH proportion (in CH_3CN) for acetamide synthesis. The error bars were drawn based on two parallel experiments.

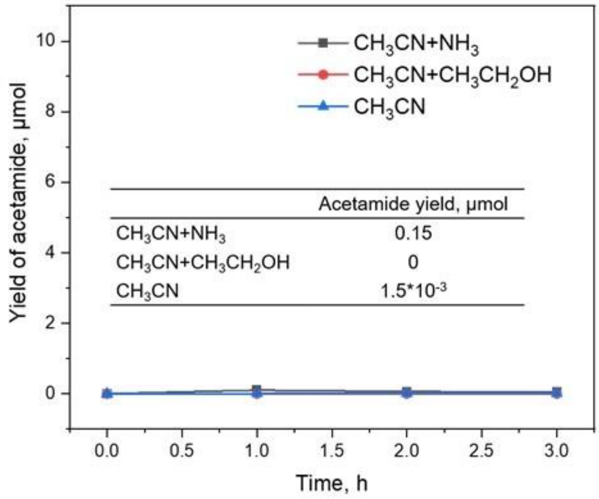

Supplementary Figure 6 | Blank experiments. Blank experiment to exclude the potential contaminant from CH_3CN hydrolysis.

Supplementary Note 16 | Excluding the CH_3CN hydrolysis for CH_3CONH_2 production

Since almost no water was included in the reaction solution, the potential hydrolysis of CH_3CN was impeded. As illustrated in Supplementary Fig. 6, trace CH_3CONH_2 (0.05 μmol h^{-1}) is generated from the CH_3CN hydrolysis, which is far below the CH_3CONH_2 production yield from the co-oxidation of CH_3CH_2OH and NH_3 (209.65 μmol h^{-1}, Supplementary Fig. 5). Moreover, no continuous accumulation of CH_3CONH_2 is observed from CH_3CN hydrolysis, which indicates that the contribution of CH_3CN solvent to CH_3CONH_2 synthesis can be neglected. The corresponding blank experiments are conducted to deduct the trace CH_3CONH_2 from CH_3CN hydrolysis (Supplementary Fig. 6).

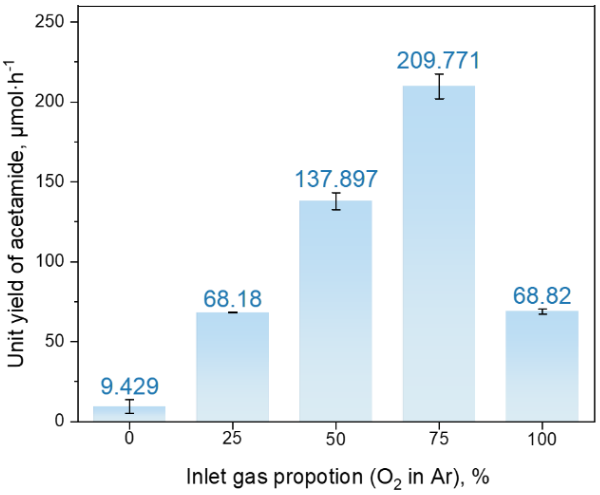

Supplementary Figure 7 | Screening experiments. Screening of the optimum O_2 proportion (in Ar) for acetamide synthesis. The error bars were drawn based on two parallel experiments.

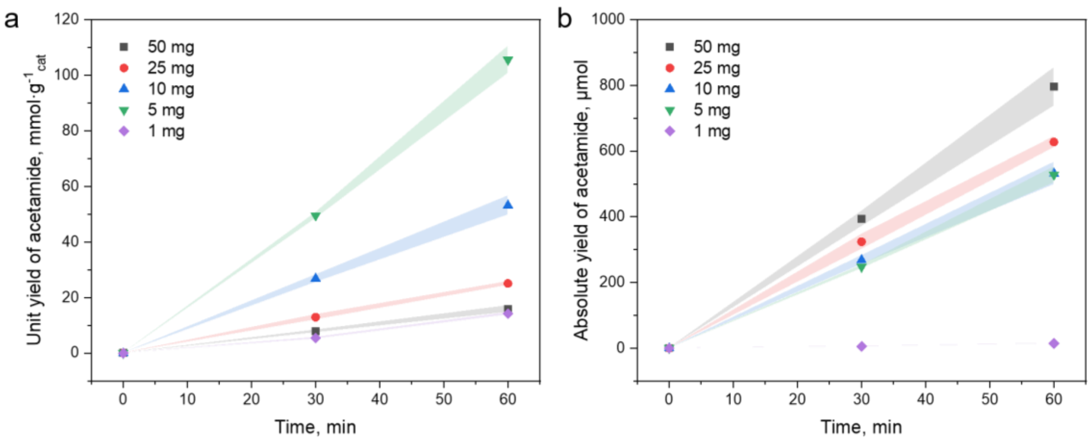

Supplementary Figure 8 | Screening experiments. Screening of the optimum catalyst dosage for acetamide synthesis. The error bars were drawn based on two parallel experiments. (a) The unit yield of different catalyst dosages for acetamide. (b) The absolute yield of different catalyst dosages for acetamide.

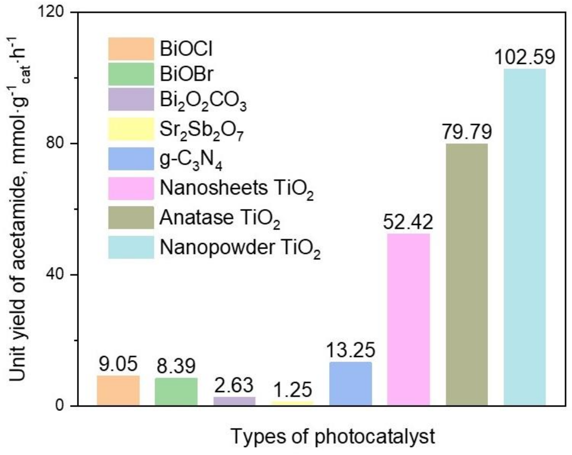

Supplementary Figure 9 | Universal experiments. C-N coupling performance of different photocatalysts.

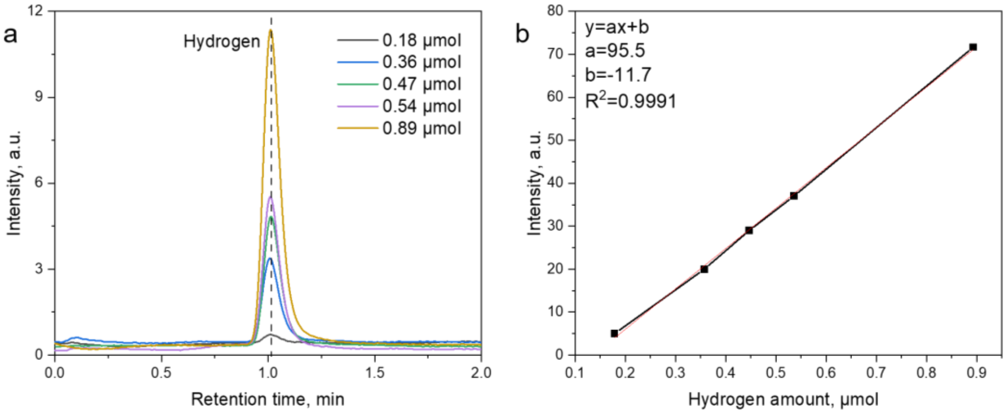

Supplementary Figure 10 | Standard curves. Tested standard intensities (a) and curves (b) for H_2 detection by a GC-TCD.

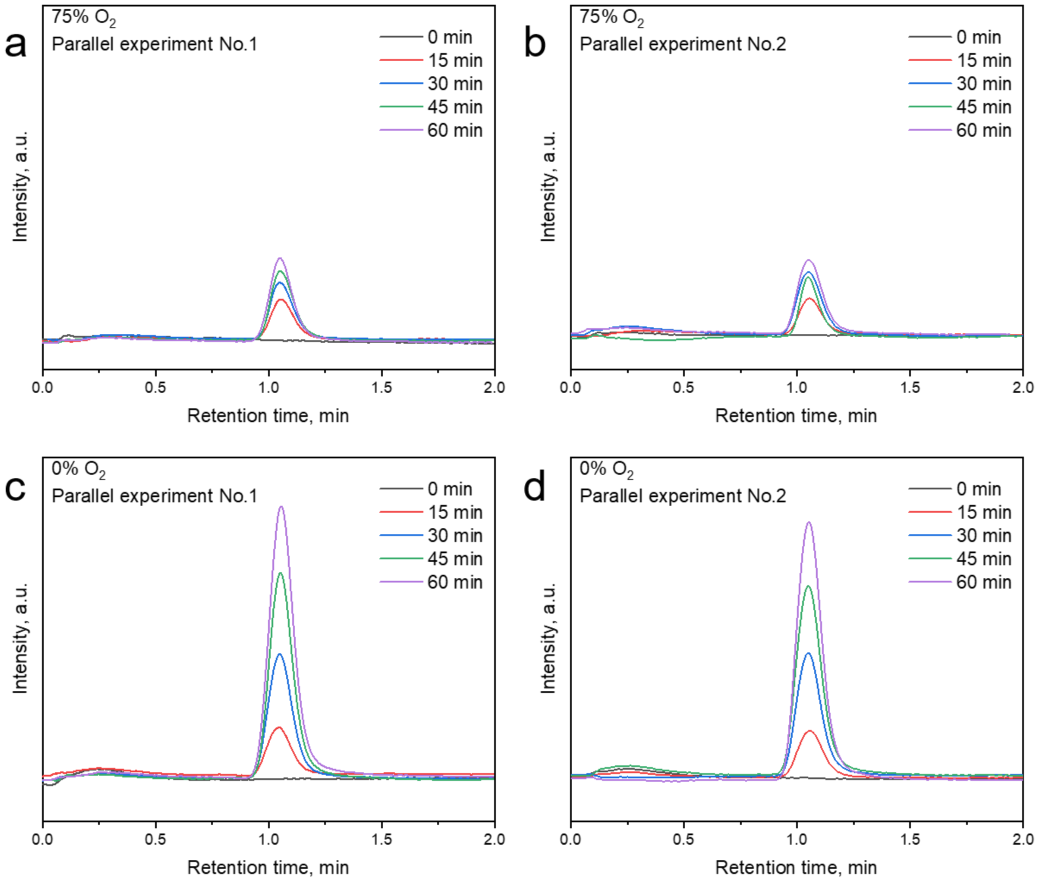

Supplementary Figure 11 | Parallel experiments. Parallel experiments for H_2 evolution under the O_2 proportion of 75% (a and b) and 0% (c and d).

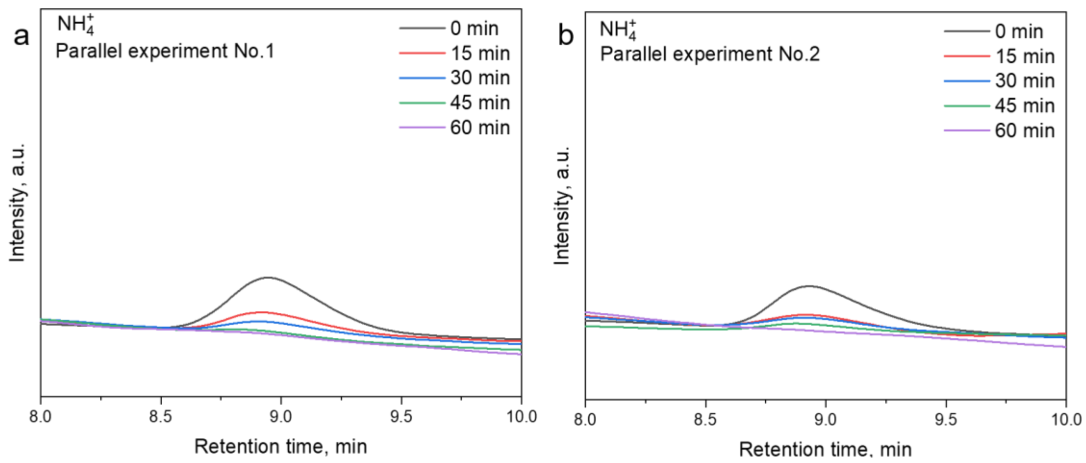

Supplementary Figure 12 | Parallel experiments. Parallel experiments for the NH_4^+ concentration (a and b) for the CH_3CONH_2 selectivity (N) test.

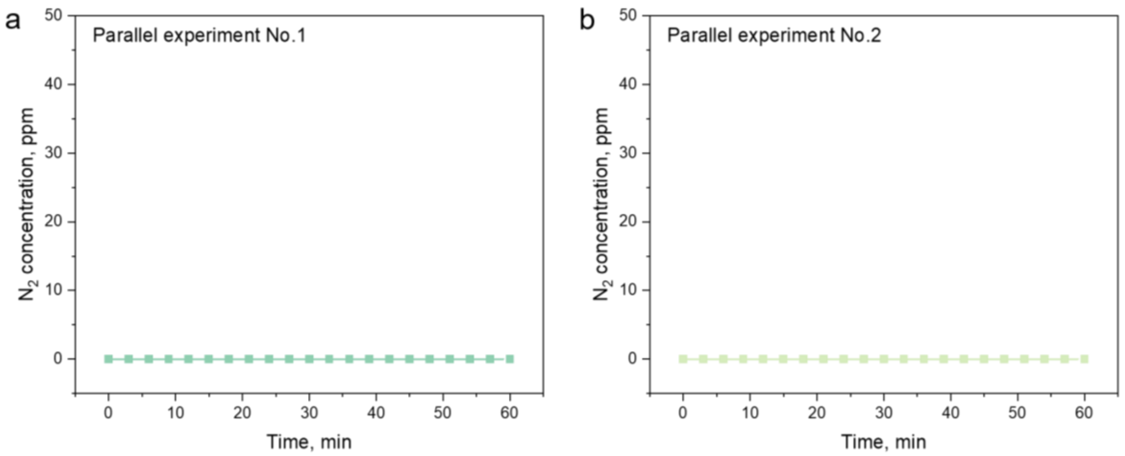

Supplementary Figure 13 | Parallel experiments. Parallel experiments for the N_2 production yield (a and b) for the CH_3CONH_2 selectivity (N) test.

Since the precise quantification of N_2 was difficult due to low N_2 yield from NH_3 peroxidation and potential contaminative N_2 in air, the N_2 yield was calculated from the N-balance to evaluate the CH_3CONH_2 selectivity regarding N-species.

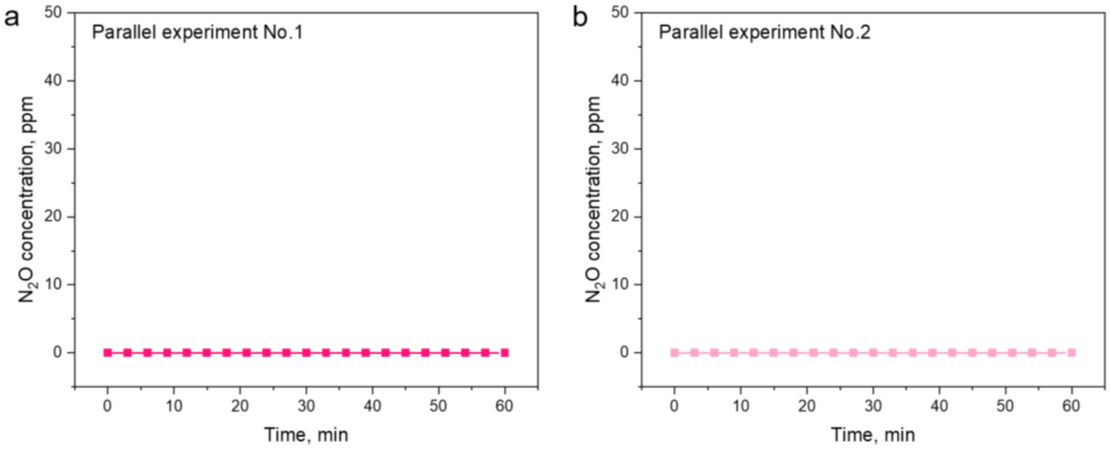

Supplementary Figure 14 | Parallel experiments. Parallel experiments for the N_2O production yield (a and b) for the CH_3CONH_2 selectivity (N) test.

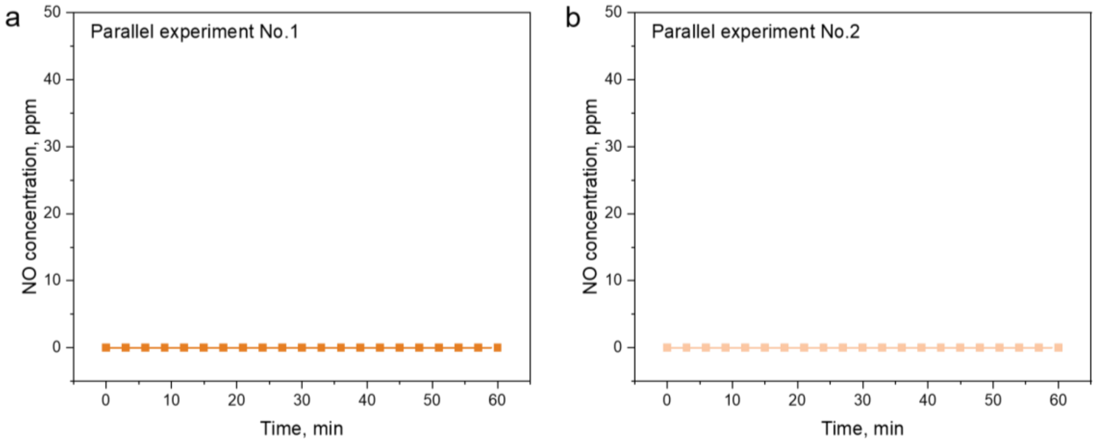

Supplementary Figure 15 | Parallel experiments. Parallel experiments for the NO production yield (a and b) for the CH_3CONH_2 selectivity (N) test.

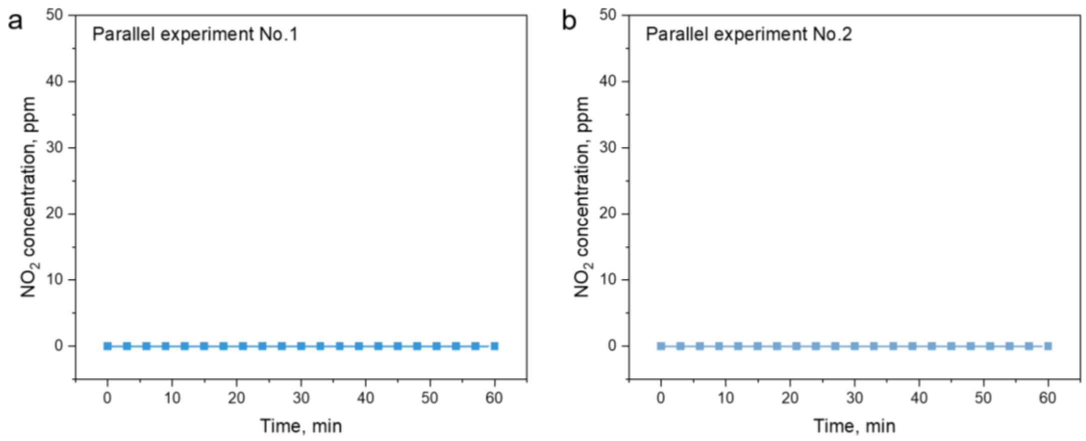

Supplementary Figure 16 | Parallel experiments. Parallel experiments for the NO_2 production yield (a and b) for the CH_3CONH_2 selectivity (N) test.

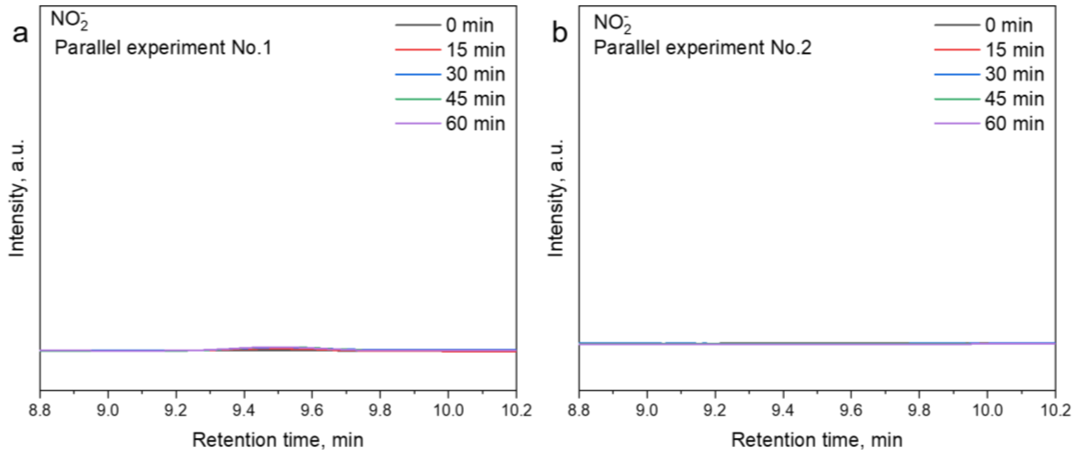

Supplementary Figure 17 | Parallel experiments. Parallel experiments for the NO_2^- concentration (a and b) for the CH_3CONH_2 selectivity (N) test.

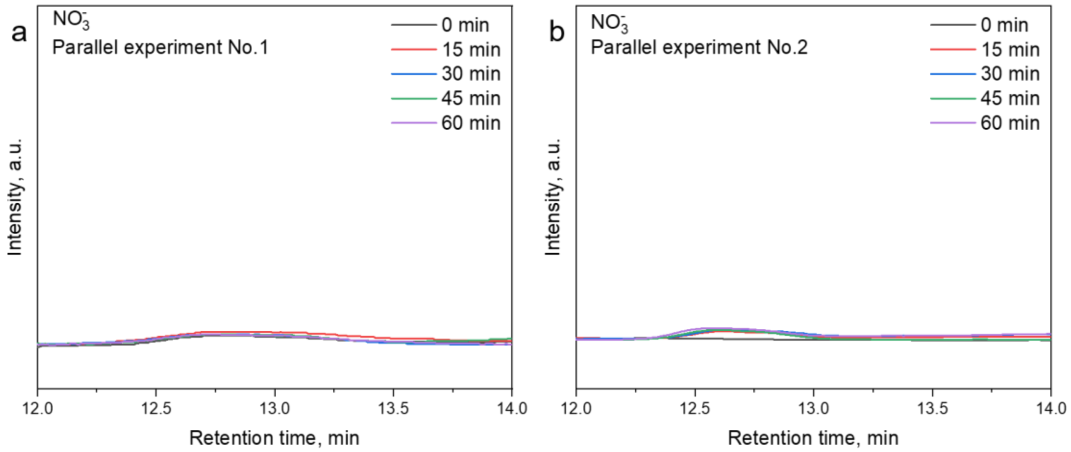

Supplementary Figure 18 | Parallel experiments. Parallel experiments for the NO_3^- concentration (a and b) for the CH_3CONH_2 selectivity (N) test.

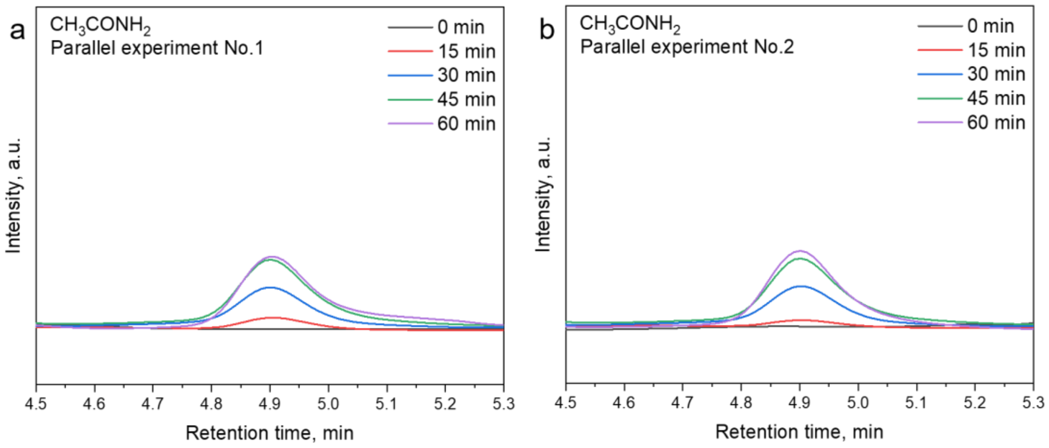

Supplementary Figure 19 | Parallel experiments. Parallel experiments for the CH_3CONH_2 concentration (a and b) for the CH_3CONH_2 selectivity (N) test.

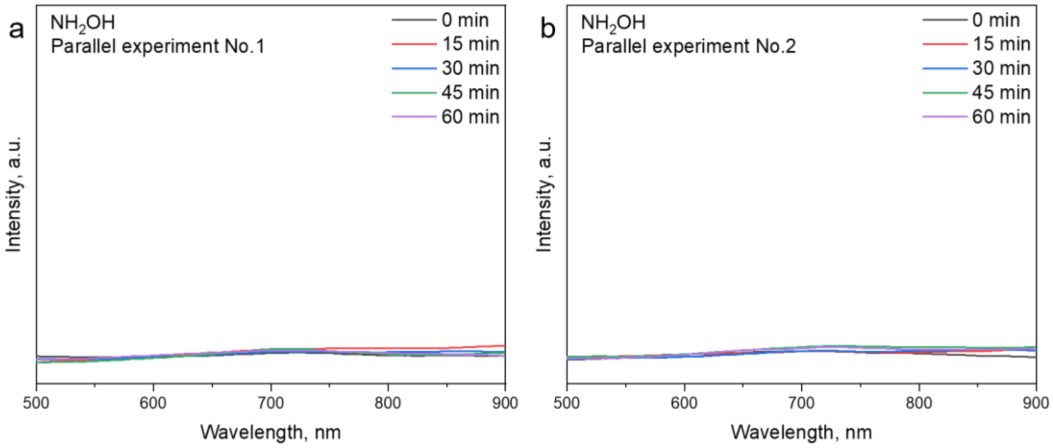

Supplementary Figure 20 | Parallel experiments. Parallel experiments for the NH_2OH (a and b) for the CH_3CONH_2 selectivity (N) test.

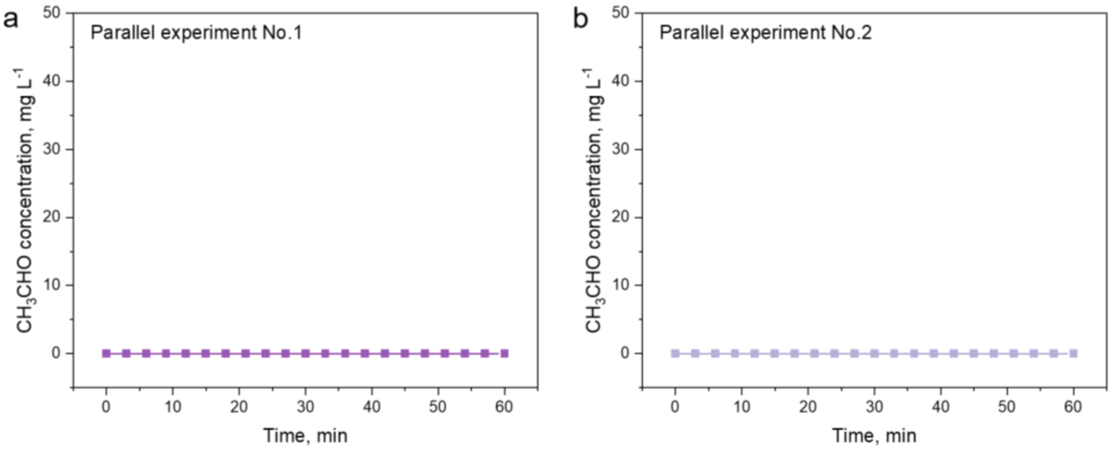

Supplementary Figure 21 | Parallel experiments. Parallel experiments for the CH_3CHO production yield (a and b) for the CH_3CONH_2 selectivity (C) test.

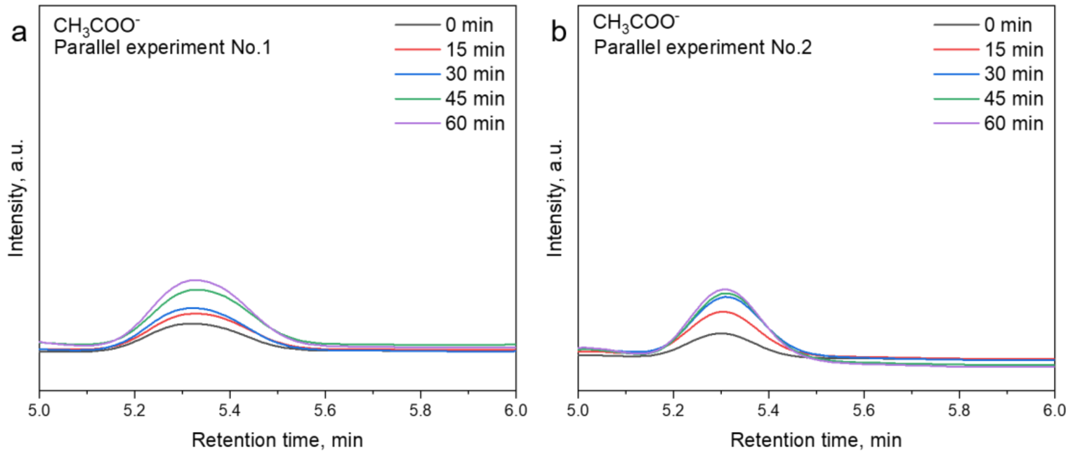

Supplementary Figure 22 | Parallel experiments. Parallel experiments for the CH_3COOH production yield (a and b) for the CH_3CONH_2 selectivity (C) test.

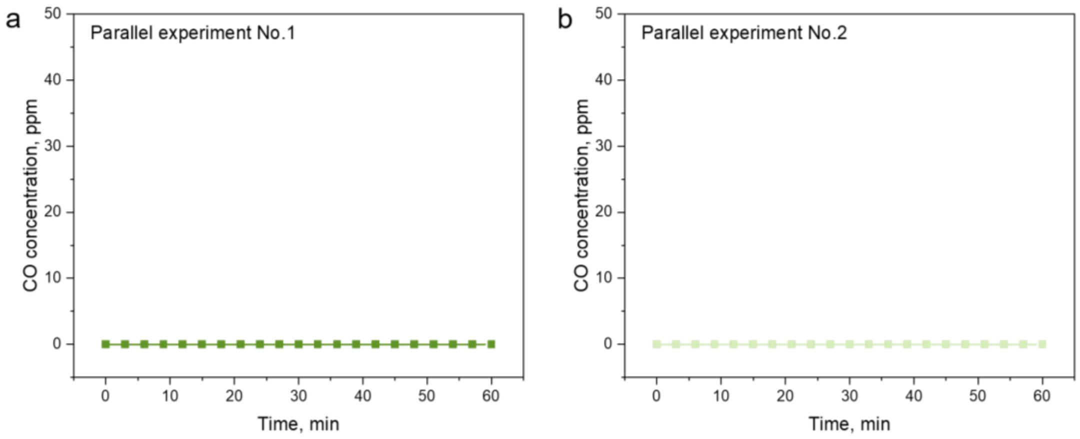

Supplementary Figure 23 | Parallel experiments. Parallel experiments for the CO production yield (a and b) for the CH_3CONH_2 selectivity (C) test.

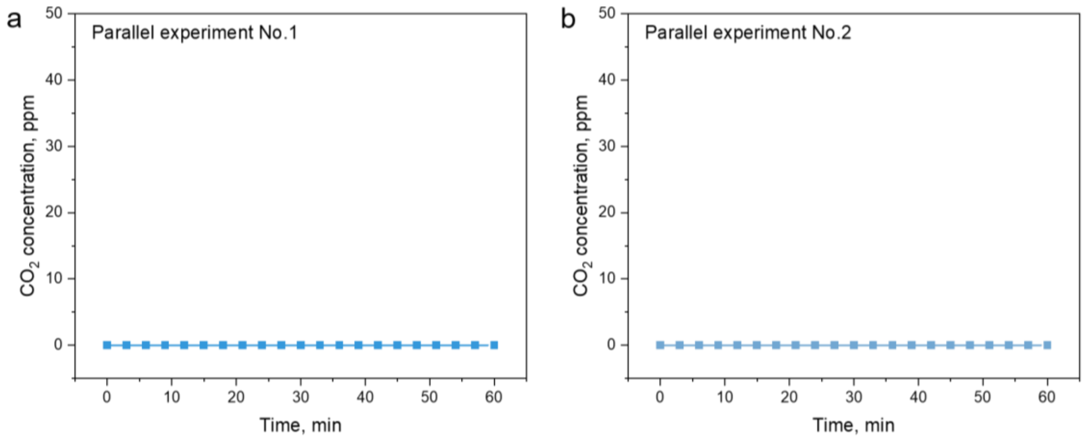

Supplementary Figure 24 | Parallel experiments. Parallel experiments for the CO_2 production yield (a and b) for the CH_3CONH_2 selectivity (C) test.

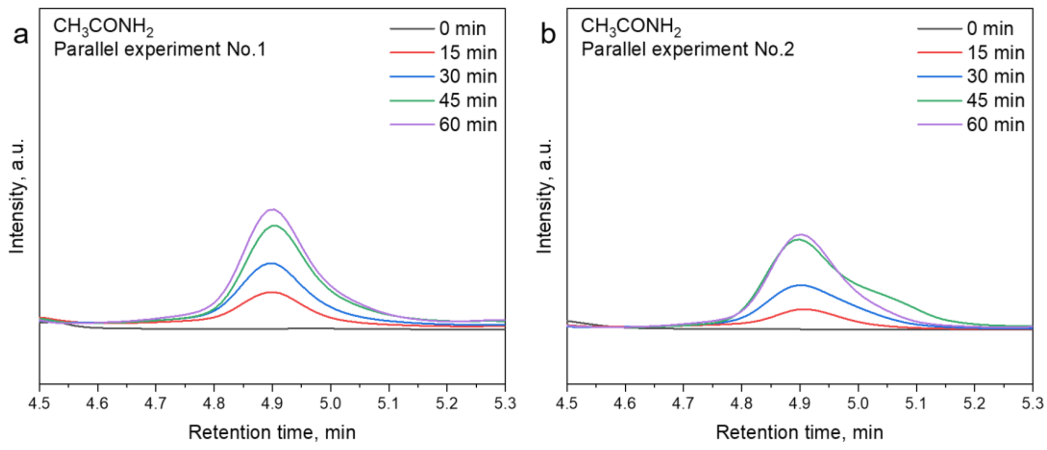

Supplementary Figure 25 | Parallel experiments. Parallel experiments for the CH_3CONH_2 (a and b) for the CH_3CONH_2 selectivity (C) test.

Supplementary Note 17 | Difference of parameters in selectivity (N/C) evaluation.

The reaction parameters for the CH_3CONH_2 selectivity evaluation towards NH_3 and CH_3CH_2OH are not consistent due to the following reasons. As depicted in Fig. 2b, the excess provision of NH_3 may lead to its peroxidation into N_2, which decreases the CH_3CONH_2 selectivity (N). Therefore, the limited NH_3 concentration (100.00 mg L^{-1}) is provided for its mild and selective oxidation into -NH_2 species for coupling. In comparison, to impede the peroxidation of CH_3CH_2OH, more NH_3 (800.00 mg L^{-1}) is included, which sufficiently consumes h^+ to avoid the peroxidation of CH_3CH_2OH into CH_3COOH. Hence the C-intermediates of -C=O can be stably accumulated. Similar results are also observed in the *in-situ* ATR-FTIR spectra (Fig. 3), in which peroxidation of individual C-/N-source proceeds without the coupled oxidative reactant. Therefore, it is reasonable that different parameters of NH_3 concentration are applied to establish objective conclusions for the selective oxidation of NH_3 and CH_3CH_2OH for CH_3CONH_2 synthesis respectively.

Supplementary Table 2 | Comparison of the photocatalytic route for the CH_3CONH_2 synthesis with the conventional route for the industrial CH_3CONH_2 synthesis.

	Photocatalytic route	Conventional route
Raw materials	$CH_3CH_2OH + NH_3$	$CH_3COOH + NH_3$
Reaction temperature	25 ℃	200 ℃
Synthesis efficiency	97.67% ± 1.67% (N-selectivity)<br>95.60%±0.22% (C-selectivity)	87%~90%
Reaction mechanism	$CH_3CH_2OH + NH_3 \rightarrow CH_3CONH_2 + 2H_2$	$CH_3COOH + NH_3 \rightarrow CH_3COONH_4$<br>$CH_3COONH_4 \rightarrow CH_3CONH_2 + H_2O$
Cost (Price in December 2023)	Ethanol: 586 USD/MT<br>Ammonia:760 USD/MT	Acetic Acid: 595 USD/MT<br>Ammonia: 760 USD/MT

Supplementary Table 3 | Comparison of the electro/photocatalytic C-N coupling efficiency from recently published literature, including the targets of production rate and selectivity (or apparent quantum yield).

Catalyst	Reaction route	C-source	N-source	Light source	Light intensity (mW cm $^{-2}$)	C-N coupling product	Production rate (mmol g_{cat}^{-1} h^{-1})	Product selectivity/	Refs.
CeO_2-500	photocatalysis	CO_2	N_2	300 W Xe lamp	Not reported	Urea	0.01	Not reported	1
Ru-TiO_2	photocatalysis	CO_2	N_2	300 W Xe lamp	10	Urea	0.025	6.3	2
Ni_1-CdS/WO_3	photocatalysis	CO_2	N_2	300 W Xe lamp	150	Urea	5.2	0.15	3
Pt cluster/TiO_2	photocatalysis	CH_3OH	N_2	300 W mercury lamp	93	Urea	0.11	97.29	4
Bi-TiO_2	photocatalysis	CH_3OH	NH_3	300 W Xe lamp	86	Formamide	5.47 ± 0.03	84.8	5
Ba-TiO_2	photocatalysis	CH_3OH	NO_3^-	365 nm light source	Not reported	Glycine	0.87	Not reported	6
Ru_1/CdS	photocatalysis	LA	NH_3	300 W Xe lamp	Not reported	Alanine	41.6	78.6	7
**TiO_2 (P25)**	**photocatalysis**	**CH_3CH_2OH**	**NH_3**	**300 W Xe lamp**	**112**	**Acetamide**	**105.61±4.86**	**99.17±0. 39**	**This work**

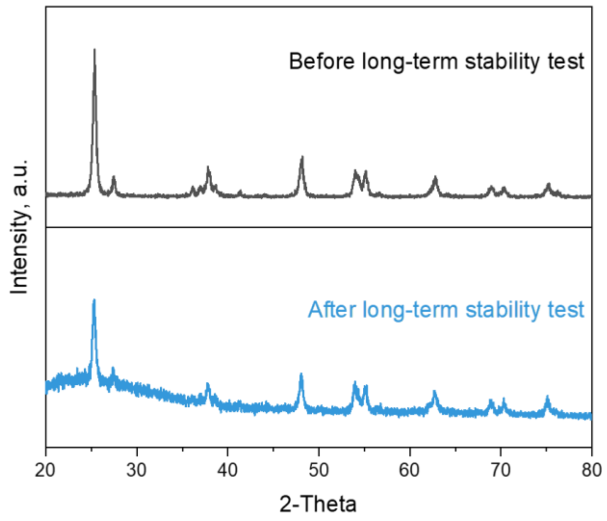

Supplementary Figure 26 | Crystalline characterizations. XRD patterns for P25 before and after the long-term stability test.

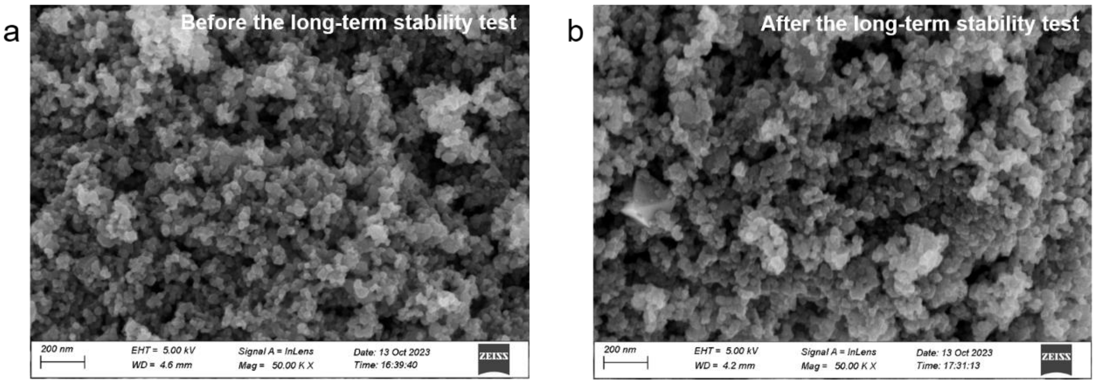

Supplementary Figure 27 | Morphology characterizations. SEM images for P25 before (a) and after (b) the long-term stability test.

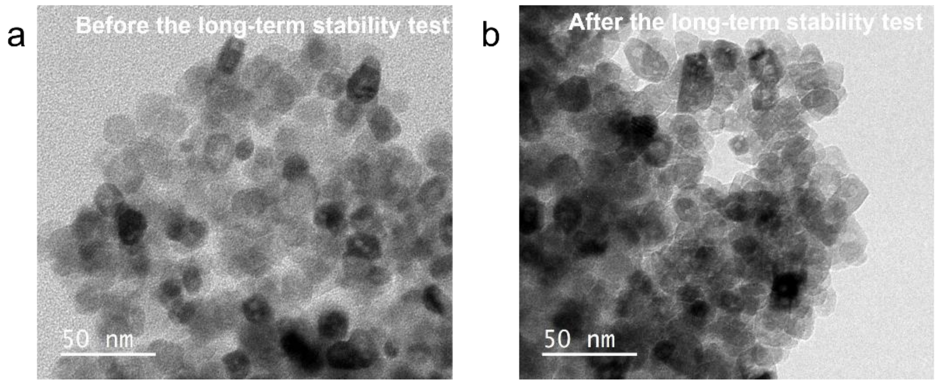

Supplementary Figure 28 | Morphology characterizations. Low-resolution TEM images for P25 before (a) and after (b) the long-term stability test.

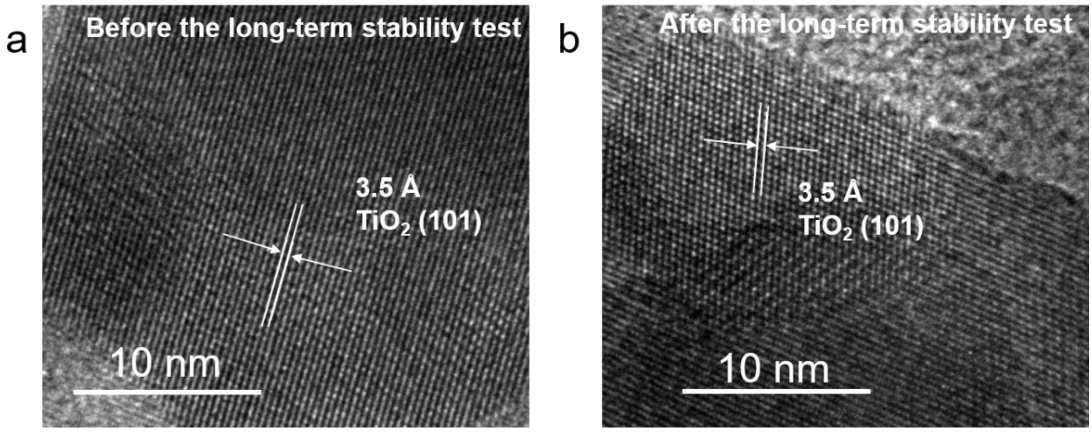

Supplementary Figure 29 | Morphology characterizations. TEM images for P25 before (a) and after (b) the long-term stability test.

Supplementary Note 18 | Characterization results of the P25 photocatalyst before and after the long-term stability.

Before and after 300 hours of the long-term stability test, the crystal structures of P25 photocatalysts remain consistent as shown in the XRD patterns (Supplementary Fig. 26). Besides, the morphological structure and the lattice stripes of P25 photocatalysts are stable, as shown by the SEM (Supplementary Fig. 27) and TEM (Supplementary Fig. 28 and 29) images.

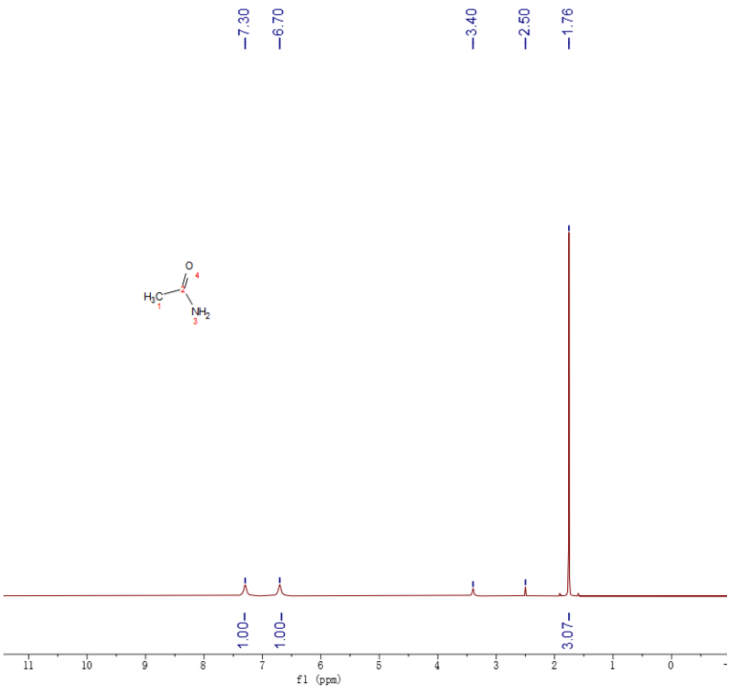

Supplementary Figure 30 | Product identification. Nuclear magnetic resonance (^{1}H NMR) result for the products after the long-term stability test.

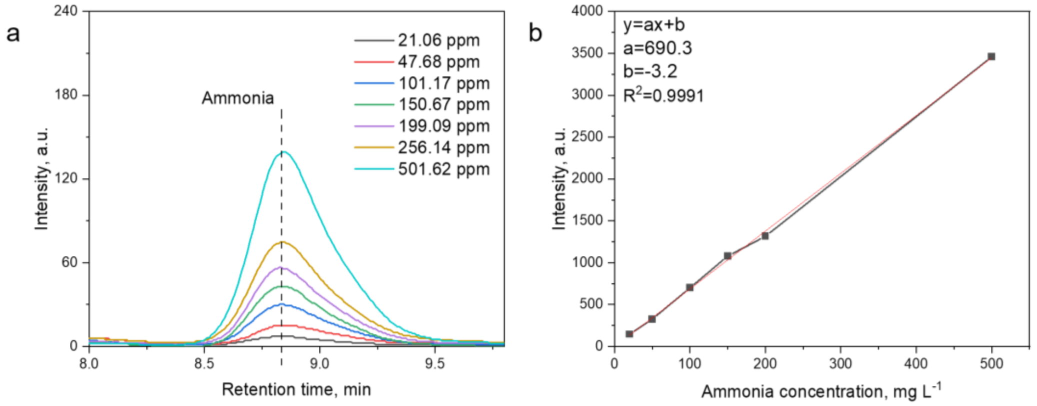

Supplementary Figure 31 | Standard curves. Tested standard intensities (a) and standard curves (b) for NH_4^+ detection by IC.

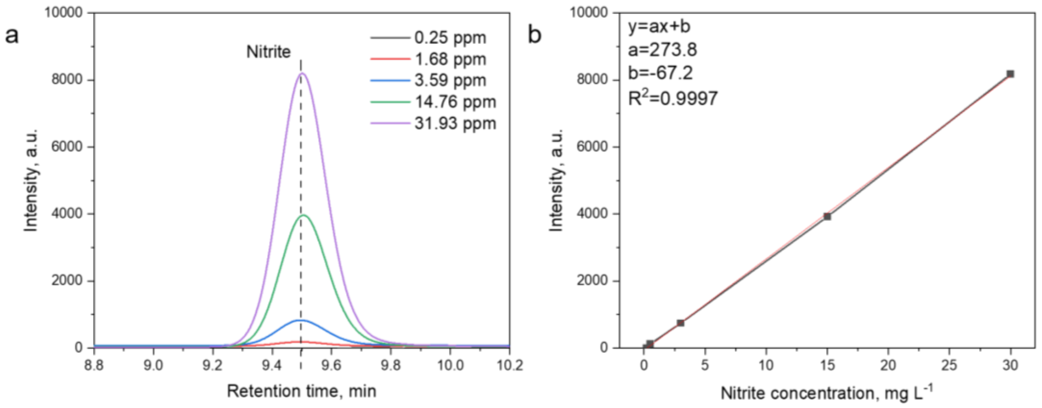

Supplementary Figure 32 | Standard curves. Tested standard intensities (a) and standard curves (b) for NO_2^- detection by IC.

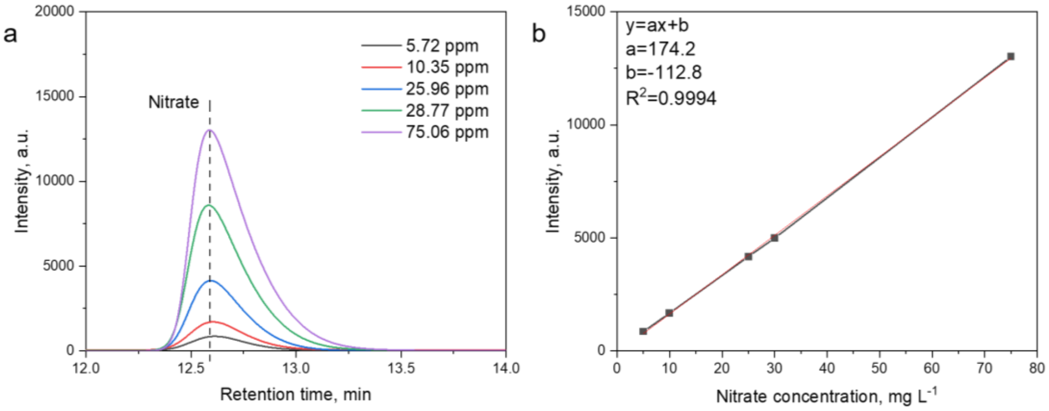

Supplementary Figure 33 | Standard curves. Tested standard intensities (a) and standard curves (b) for NO_3^- detection by IC.

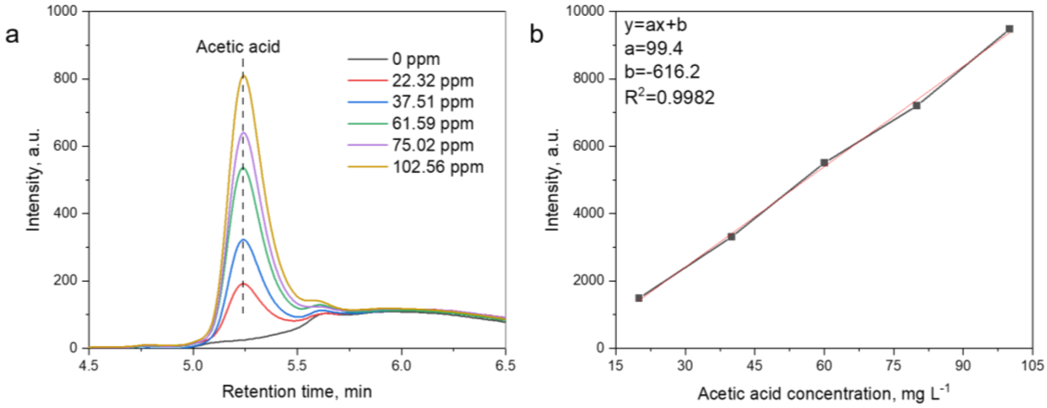

Supplementary Figure 34 | Standard curves. Tested standard intensities (a) and standard curves (b) for CH_3COOH detection by IC.

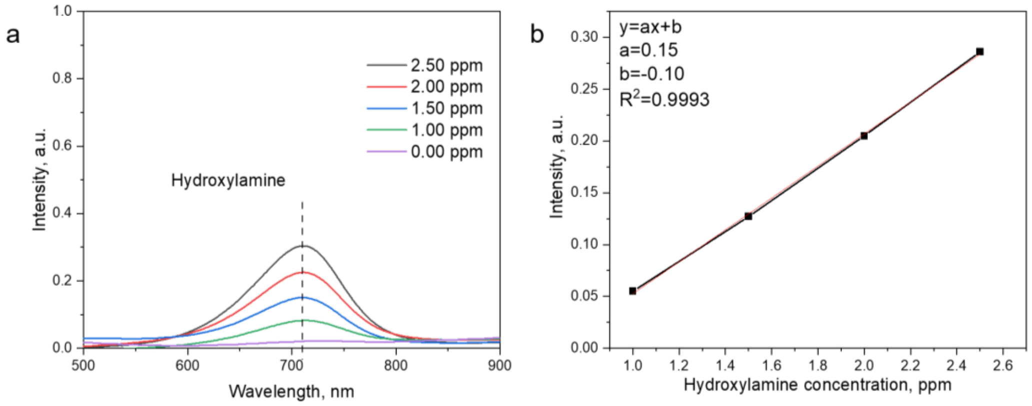

Supplementary Figure 35 | Standard curves. Tested standard intensities (a) and curves (b) for NH_2OH detection by a UV-vis.

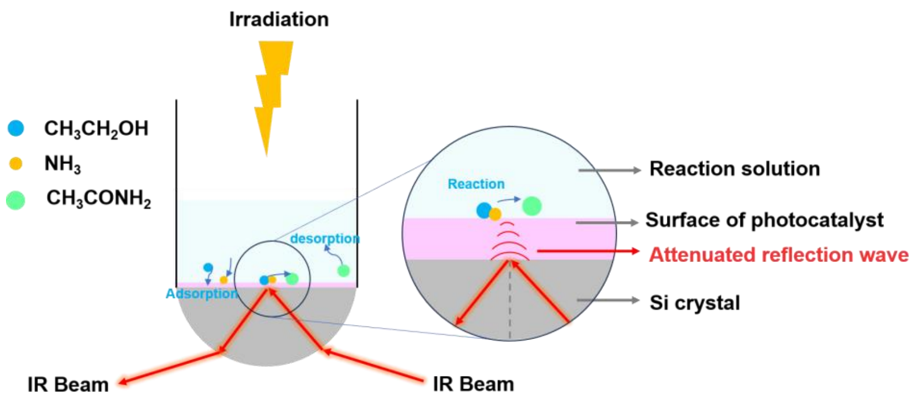

Supplementary Figure 36 | Schematic diagram. Illustrating the principle of the *in-situ* liquid ATR FT-IR.

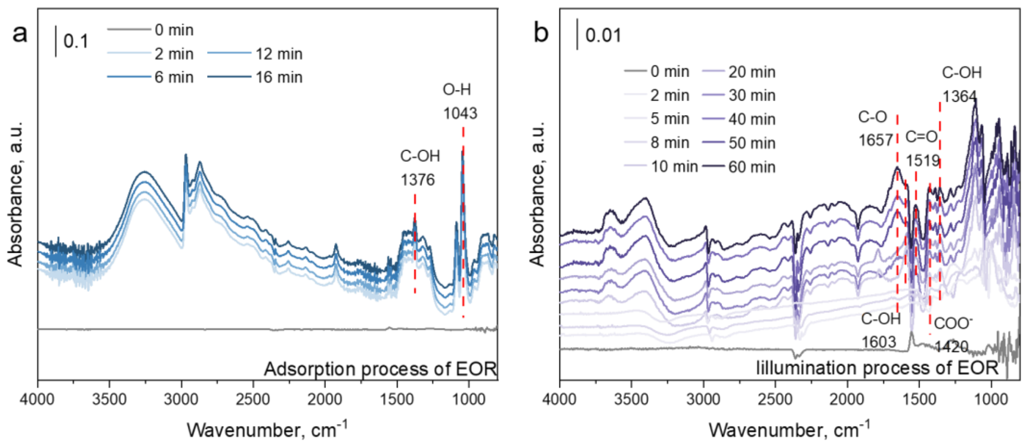

Supplementary Figure 37 | *In-situ* ATR-FTIR results. *In-situ* ATR-FTIR results for the adsorption in dark (a) and photocatalysis (b) process of individual EOR.

Supplementary Table 4 | Assignments of the IR bands in the absorption and photocatalysis process of individual EOR.

Wavenumber, cm^{-1}	Assignment	Refs.
1657	C-O	8
1603, 1376 and 1364	C-OH	9
1519	C=O	10
1420	COO^{-}	11
1043	O-H	9

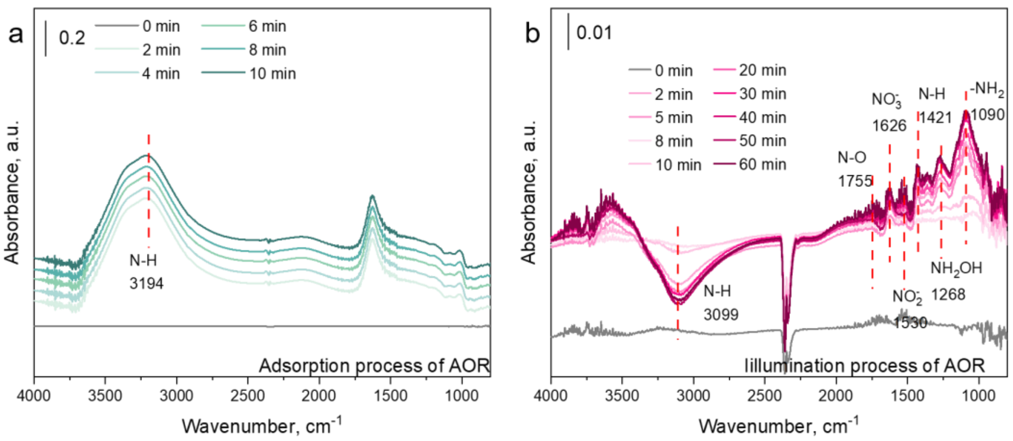

Supplementary Figure 38 | *In-situ* ATR-FTIR results. *In-situ* ATR-FTIR results for the adsorption in dark (a) and photocatalysis (b) process of individual AOR.

Supplementary Table 5 | Assignments of the IR bands in the absorption and photocatalysis process of individual AOR.

Wavenumber, cm^{-1}	Assignment	Refs.
3194, 3099 and 1421	N-H	12
1755	N-O	13
1626	NO_3^-	14
1530	NO_2^-	15
1268 and 1244	NH_2OH	16
1090	$-NH_2$	17

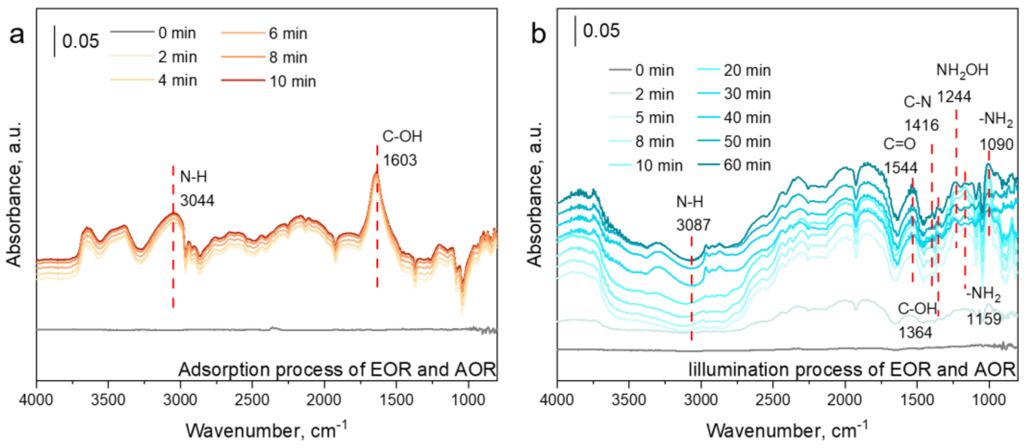

Supplementary Figure 39 | *In-situ* ATR-FTIR results. *In-situ* ATR-FTIR results for the adsorption in dark (a) and photocatalysis (b) process of combined EOR and AOR.

These figures (Supplementary Figures 37-39) can be divided into two reaction processes of adsorption in the dark (Supplementary Figures 37a, 38a and 39a) and photocatalysis under light irradiation (Supplementary Figures 37b, 38b and 39b). Here are the explanations of the intensity variation in these two processes respectively.

1. Adsorption process in dark.

Under dark conditions, the data curve at 0 min refers to the initial infrared signal on the catalyst surface. To achieve precise tracking of adsorbed N and C substances, we have subtracted the catalyst surface signal as the background, resulting in a relatively flat curve. After the reaction is drop-coated on the catalyst surface, the reactive substances rapidly adsorb on the catalyst surface, causing a significant change within the first 0-2 mins. The characteristic peaks are attributed to reactive species such as C and N.

Supplementary Figure 37a: 1376 C-OH (1376 cm^{-1}) and O-H (1043 cm^{-1}) species.

Supplementary Figure 38a: N-H (3194 cm^{-1}) species.

Supplementary Figure 39a: C-OH (1603 cm^{-1}) and N-H (3044 cm^{-1}) species.

After 2 min, there is a continuous accumulation process of surface functional groups. Hence, the intensity of the characteristic peaks of the species will increase without the appearance of new substances. As explained in the *Response to Comment 3*, once the adsorption-desorption equilibrium is reached and the sites are fully occupied, the intensity of the characteristic curves tends to stabilize.

2. Photocatalysis process under light irradiation.

Under light irradiation, the signals at 0 min represent the result of subtracting the adsorption equilibrium curve as the background before turning on the light, which is the initial infrared signal of the reaction catalyst after adsorbing the reactants to equilibrium, which is similarly quite flat with that at the dark condition. Under light exposure, the photocatalytic reaction begins, with reactants being rapidly consumed and intermediate and final products accumulating, hence there is a significant change within the first 0-2 mins.

Supplementary Figure 37b: decrease of C-OH (1603 cm^{-1} and 1364 cm^{-1}) species, increase of C=O (1519 cm^{-1}), C-O (1657 cm^{-1}) and COO^- (1420 cm^{-1}).

Supplementary Figure 38b: decrease of N-H (3099 cm^{-1}) species, increase of $-NH_2$ (1090 cm^{-1}), N-O (1755 cm^{-1}), NO_2^- (1530 cm^{-1}) and NO_3^- (1626 cm^{-1}) species.

Supplementary Figure 39b: decrease of N-H (3087 cm^{-1}) and C-OH (1364 cm^{-1}) species, increase of $-NH_2$ (1090 cm^{-1}), N=O (1544 cm^{-1}) and C-N (1416 cm^{-1}).

After 2 min, the final products continue to accumulate while reactants and some intermediate products are consumed. Subsequently, the adsorption-desorption of species on the catalyst surface reaches equilibrium, with no significant changes in peak intensity.

Supplementary Table 6 | Assignments of the IR bands in the absorption and photocatalysis process of combined EOR and AOR.

Wavenumber, cm^{-1}	Assignment	Refs.
1603 and 1364	C-OH	9
1544	C=O	10
1416	C-N	18
3087 and 3044	N-H	12
1244	NH_2OH	17
1159 and 1090	$-NH_2$	19

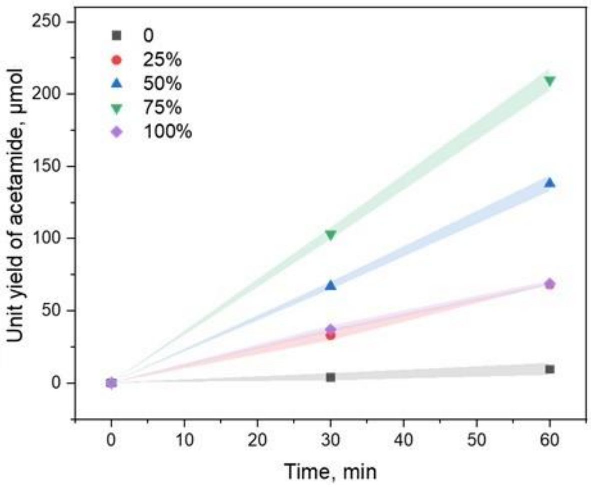

Supplementary Figure 40 | Screening experiments. Efficiency test for the O_2 proportion (in Ar) dependent CH_3CONH_2 yield. The error bars were drawn based on two parallel experiments.

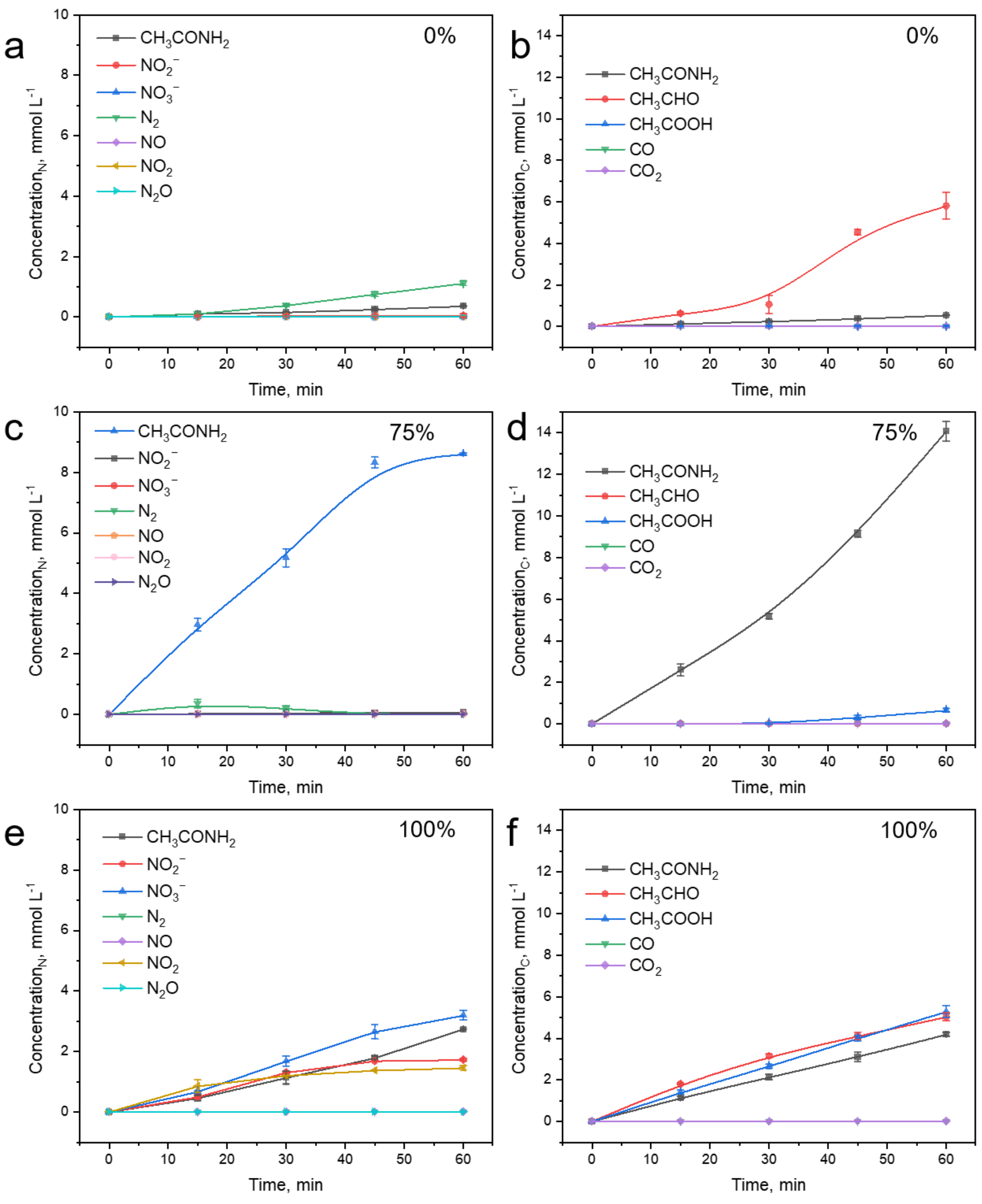

Supplementary Figure 41 | Product distribution experiments. Products concentration of photocatalytic C-N coupling at different oxygen concentrations. (a) and (b) under the O_2 proportion of 0%; (c) and (d) under the O_2 proportion of 75%; (e) and (f) under the O_2 proportion of 100%.

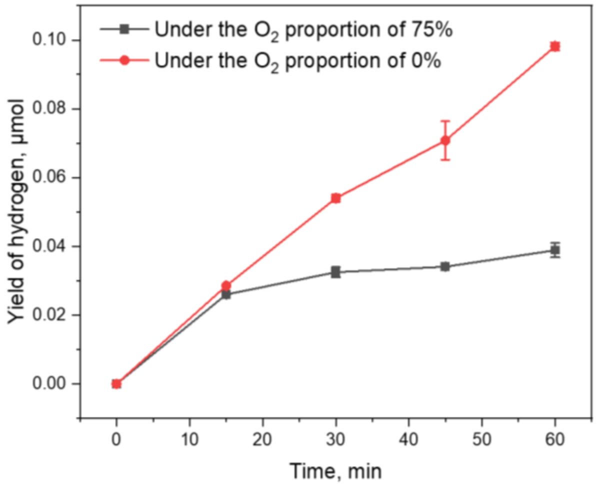

Supplementary Figure 42 | H_2 evolution experiments. Comparison between the efficiencies for H_2 evolution under the O_2 proportion of 0% and 75%. The error bars were drawn based on two parallel experiments.

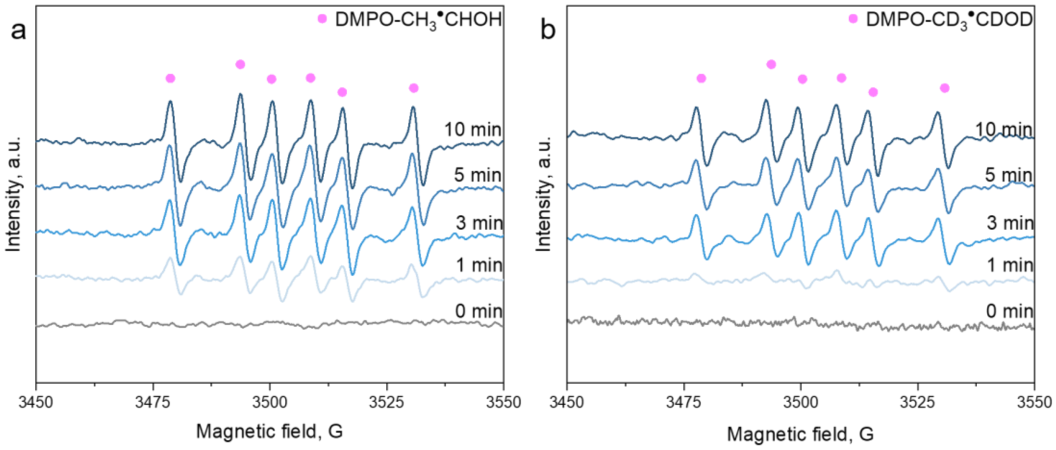

Supplementary Figure 43 | *In-situ* EPR results. *In-situ* EPR results for the detection of DMPO-$CH_3{}^{\bullet}CHOH$ (a) and DMPO-$CD_3{}^{\bullet}CDOD$ (b) respectively.

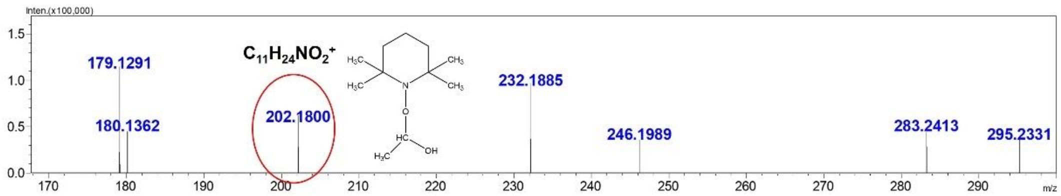

Supplementary Figure 44 | Trapping experiments. High-resolution mass spectra (HR-MS) result for the TEMPO trapped $CH_3{}^{\bullet}CHOH$.

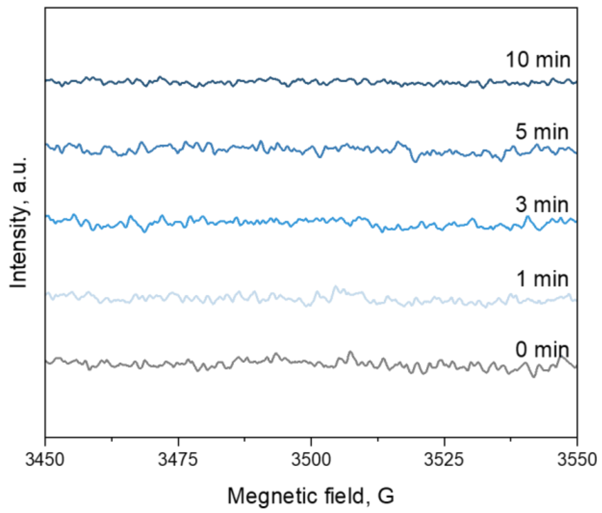

Supplementary Figure 45 | *In-situ* EPR results. *In-situ* EPR results for the detection of TEMPO from the generation of TEMP oxidation by $^1O_2$ at the O_2 proportion of 0%.

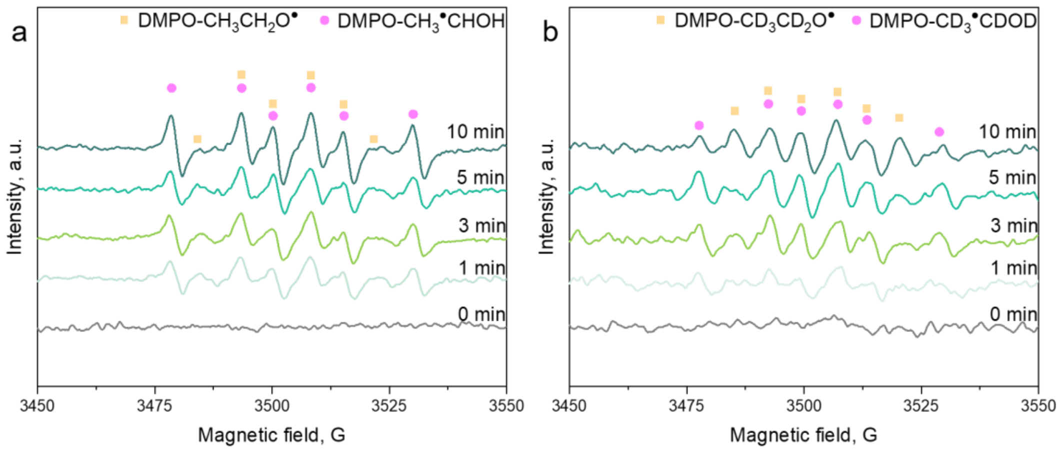

Supplementary Figure 46 | *In-situ* EPR results. *In-situ* EPR results for the detection of DMPO-$CH_3CH_2O^{\bullet}$ (a) and DMPO-$CD_3CD_2O^{\bullet}$ (b) respectively.

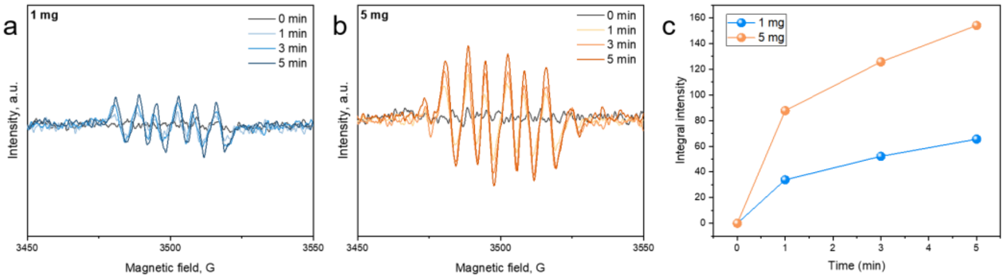

Supplementary Figure 47 | *In-situ* EPR results. *In-situ* EPR analysis for detecting DMPO-$CH_3CH_2O^{\bullet}$ at varying catalyst dosages: (a) 1 mg, (b) 5 mg, and (c) integrated signal intensity comparison for 1 mg and 5 mg.

Supplementary Note 19 | Difference of the detected signals between $CH_3{}^{\bullet}CHOH/CH_3CH_2O^{\bullet}$ and $CH_3{}^{\bullet}CHOH/CD_3CD_2O^{\bullet}$ by the DMPO-trapping EPR experiments.

The detection of DMPO-trapped alkyl and alkoxyl radicals is identified as C-centered and O-centered species respectively. Hence, the difference between these H/D labeled radicals can not be directly indexed in the EPR measurements. The corresponding MS and NMR results are thereby provided to distinguish the H/D labeled reaction species (Supplementary Figs. 37, 44-47 and Supplementary Notes 18 and 19).

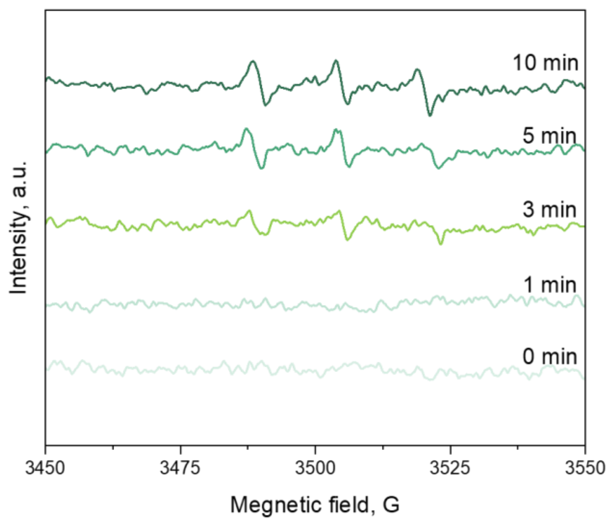

Supplementary Figure 48 | *In-situ* EPR results. *In-situ* EPR results for the detection of TEMPO from the generation of TEMP oxidation by $^1O_2$ at the O_2 proportion of 75%.

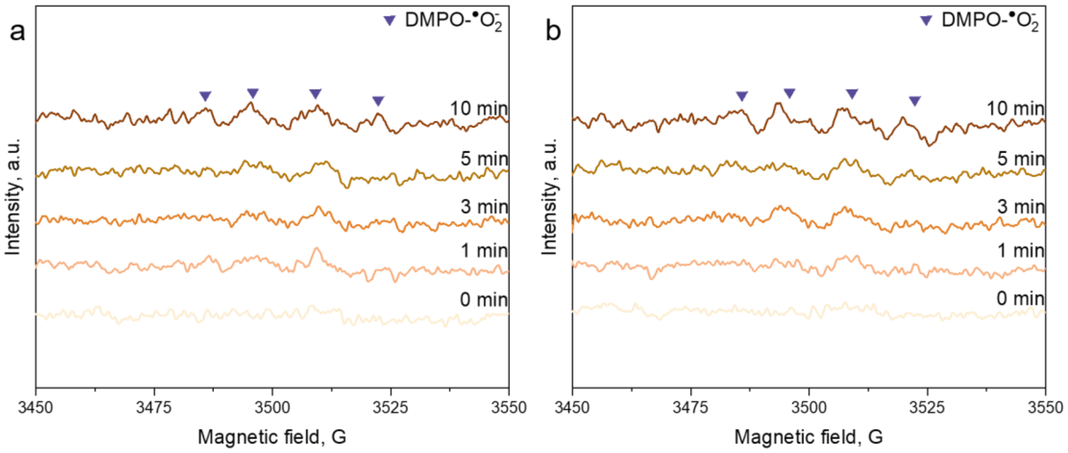

Supplementary Figure 49 | *In-situ* EPR results. *In-situ* EPR results for the detection of DMPO-$^{\bullet}O_2^-$ by using CH_3CH_2OH (a) and CD_3CD_2OD (b) as the C-sources respectively.

Supplementary Note 20 | The absence of detection of alkyl and alkoxyl radicals at the O_2 proportion of 100%.

Under the O_2 proportion of 100%, the interaction between $^{\bullet}O_2^{-}$ and DMPO agent is intensive, which impedes the detection of the alkyl and alkoxyl radicals. Moreover, as illustrated in Supplementary Fig. 34, the higher O_2 proportion contributes to the peroxidation of CH_3CH_2OH and reduces the C-N coupling efficiency. Therefore, the rapid transformation of the intermediated alkyl and alkoxyl radicals also leads to the absence of corresponding DMPO-trapped signals for $CH_3{}^{\bullet}CHOH/CH_3CH_2O^{\bullet}$ and $CH_3{}^{\bullet}CHOH/CD_3CD_2O^{\bullet}$ at such a high O_2 proportion.

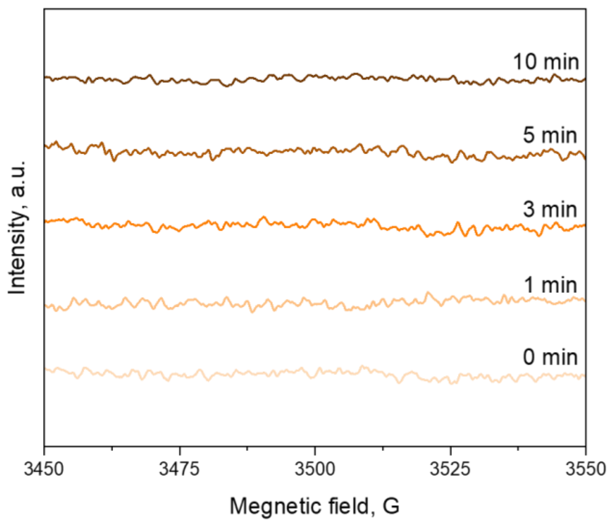

Supplementary Figure 50 | *In-situ* EPR results. *In-situ* EPR results for the detection of TEMPO from the generation of TEMP oxidation by $^{1}O_2$ at the O_2 proportion of 100%.

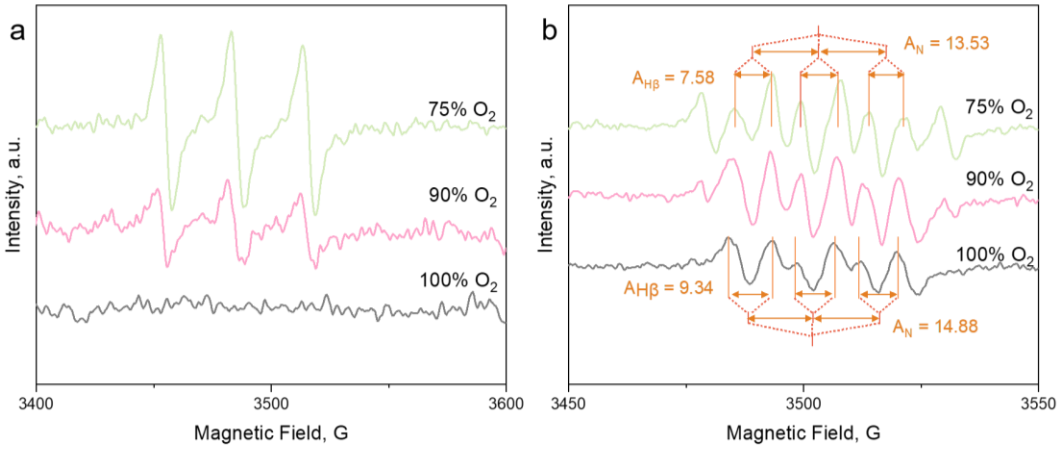

Supplementary Figure 51 | *In-situ* EPR results. *In-situ* EPR results for (a) TEMP-trapping and (b) DMPO-trapping under the O_2 proportion of 75%, 90% and 100% (d) respectively.

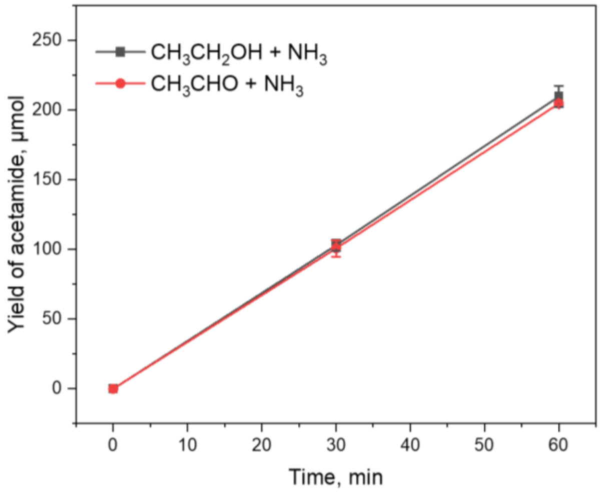

Supplementary Figure 52 | Intermediate experiments. Efficiency test for CH_3CONH_2 production by using CH_3CH_2OH and CH_3CHO as the C-sources respectively. The error bars were drawn based on two parallel experiments.

Supplementary Table 7 | Comparison of CH_3CH_2OH and CH_3CHO as reaction materials.

	Price, USD/MT	Purity	stability
CH_3CHO	1430	40% aqueous solution of acetaldehyde	unstable
CH_3CH_2OH	586	99.5% ethanol absolute	stable

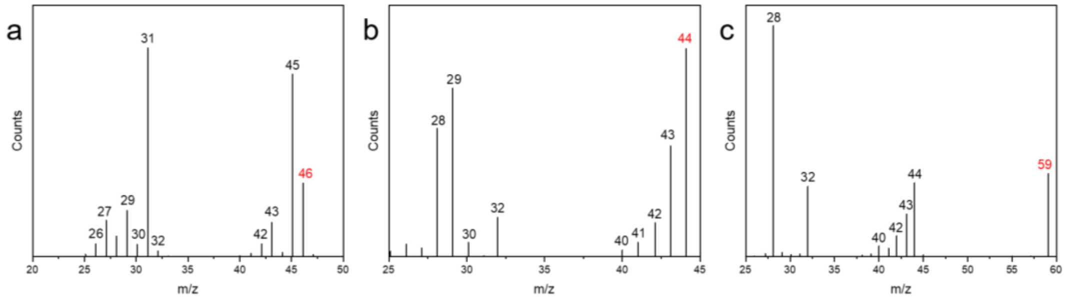

Supplementary Figure 53 | MS results. MS results for the detection of CH_3CH_2OH (a), CH_3CHO (b) and CH_3CONH_2 (c) respectively.

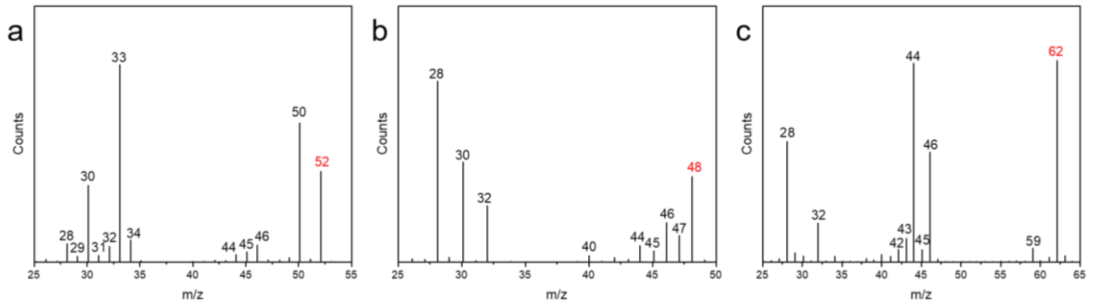

Supplementary Figure 54 | MS results. MS results for the detection of CD_3CD_2OD (a), CD_3CDO (b) and CD_3CONH_2 (c) respectively.

Supplementary Note 21 | m/z assignments of ethanol, acetaldehyde and acetamide.

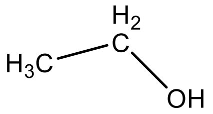

Chemical Formula: C_2H_6O; Molecular Weight: 46.0690; m/z: 46.0419;

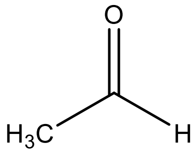

Chemical Formula: C_2H_4O; Molecular Weight: 44.0530; m/z: 44.0262;

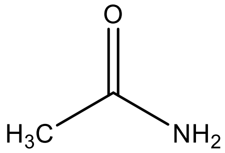

Chemical Formula: C_2H_5NO; Molecular Weight: 59.0680; m/z: 59.0371;

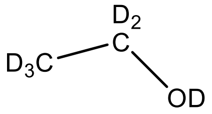

Chemical Formula: C_2D_6O; Molecular Weight: 52.1056; m/z: 52.0795;

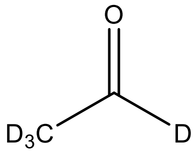

Chemical Formula: C_2D_4O; Molecular Weight: 48.0774; m/z: 48.0513;

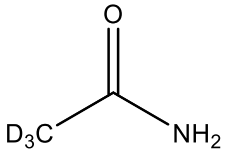

Chemical Formula: $C_2H_2D_3NO$; Molecular Weight: 62.0863; m/z: 62.0559.

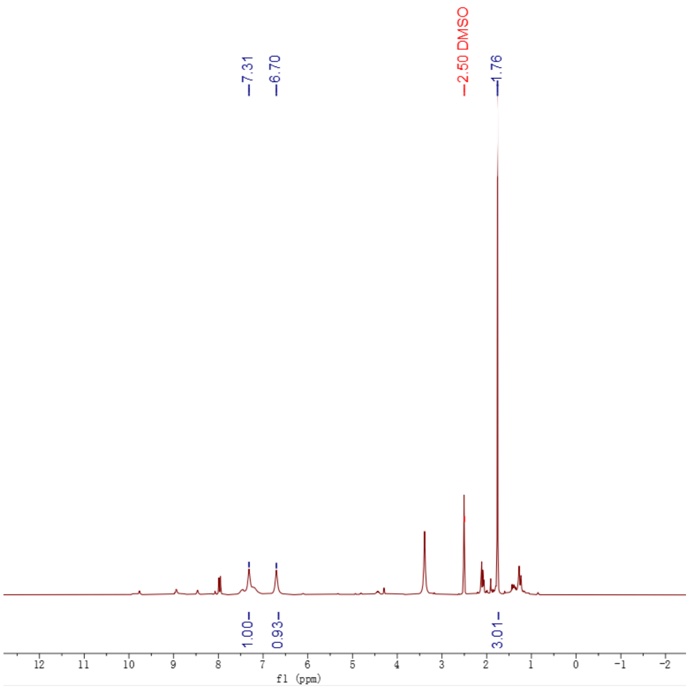

Supplementary Figure 55 | Product identification. Nuclear magnetic resonance (^{1}H NMR) result for the detection of CH_3CONH_2.

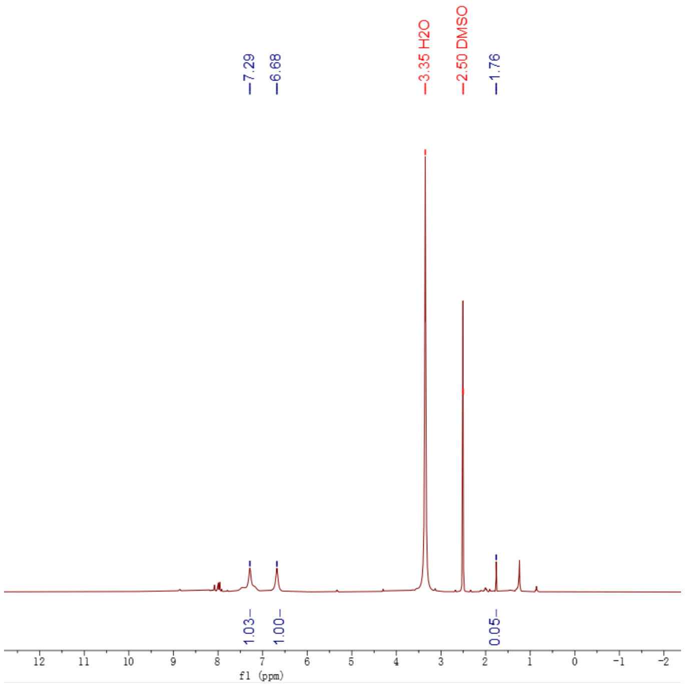

Supplementary Figure 56 | Isotopic product identification. ^{1}H NMR result for the detection of CD_3CONH_2.

Supplementary Note 22 | Chemical shift of acetamide.

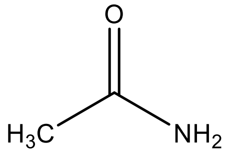

Chemical formula: C_2H_5NO;

^{1}H NMR (400 MHz, DMSO-d6): δ 7.31 (s, 1H), 6.70 (s, 1H), 1.76 (s, 3H);

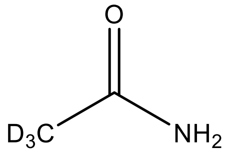

Chemical formula: $C_2H_2D_3NO$;

^{1}H NMR (400 MHz, DMSO-d6): δ 7.29 (s, 1H), 6.68 (s, 1H).

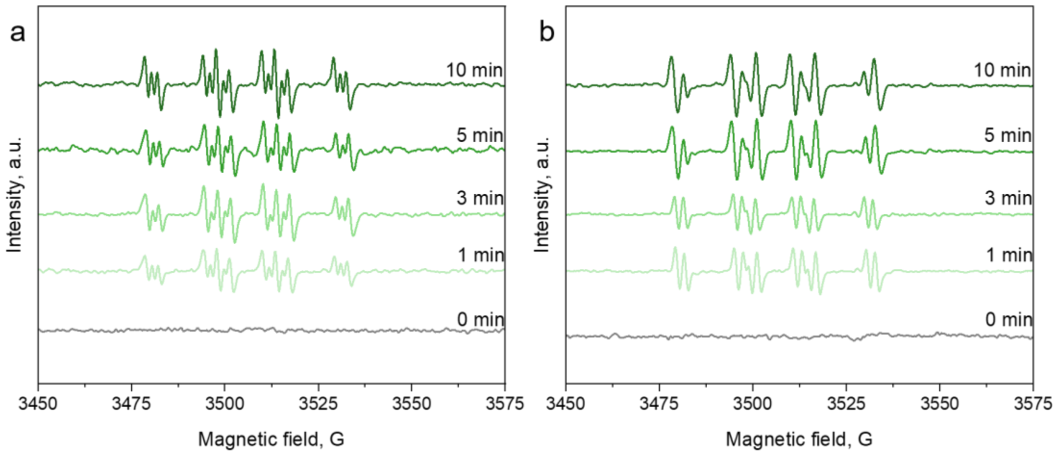

Supplementary Figure 57 | *In-situ* EPR results. *In-situ* EPR results for the detection of DMPO-$^{\bullet 14}NH_2$ (a) and DMPO-$^{\bullet 15}NH_2$ (b) respectively.

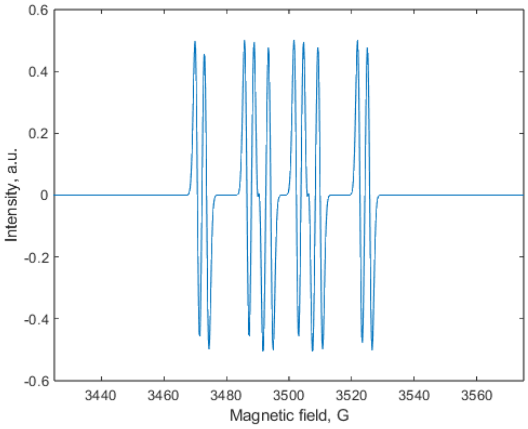

Supplementary Figure 58 | Simulation curve. Simulated EPR result for the DMPO-•$^{15}NH_2$.

Supplementary Note 23 | Applied calculation codes for the simulation of DMPO-•$^{15}NH_2$ in the software of MATLAB R2019b.

```
clear,clc,clf
[x0,y0] = eprload('NH2.dsc');
y0 = y0/max(y0);
Exp.mwFreq = 9.825865;
Exp.Range = [345 355];
Sys.g = 2.0063;
Sys.lw = 0.09;
[x,y] = garlic(Sys,Exp);
y = (y/max(y))';
Sys.g = 2.0063;
Sys.Nucs = '1H,14N,15N';
Sys.A = [3 15.45 19.9]*1.44
[x2,y2] = garlic(Sys,Exp);
y2 = 0.5*(y2/max(y2))';
x = 10*x';
x2 = 1*x0
plot(x2,y2);
```

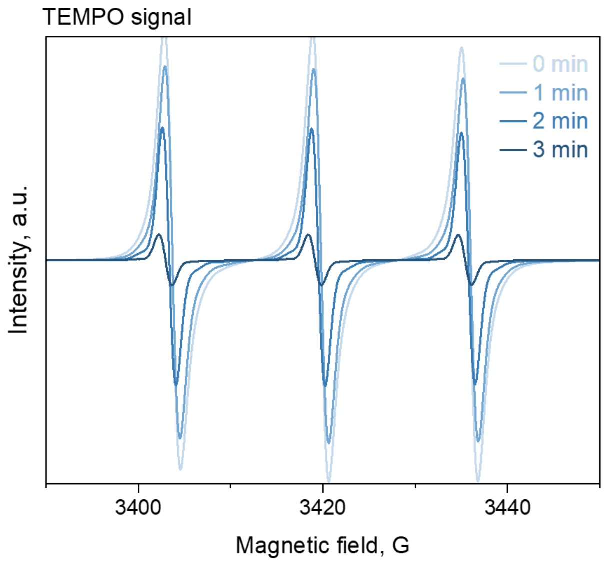

Supplementary Figure 59 | *In-situ* EPR results. *In-situ* EPR results for the detection of TEMPO reduction by light-generated e^{-} without NH_3 oxidation cooperation.

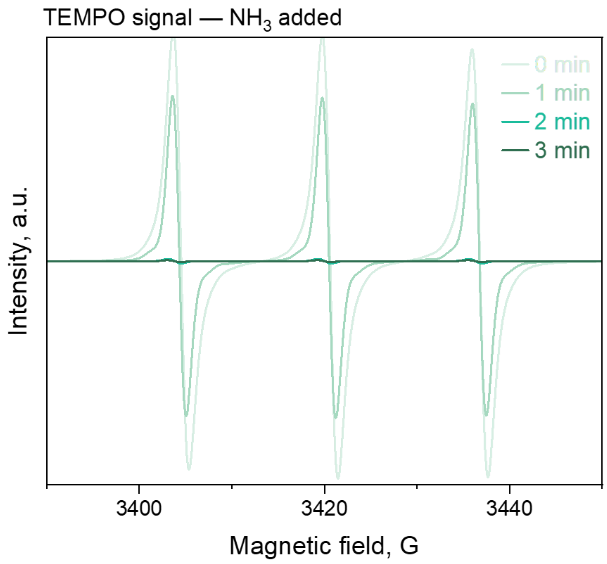

Supplementary Figure 60 | *In-situ* EPR results. *In-situ* EPR results for the detection of TEMPO reduction by light-generated e⁻ with NH_3 oxidation cooperation.

Supplementary Note 24 | details for the $^{\bullet}NH_2$ detection.

The $^{\bullet}NH_2$ is detected by DMPO-trapping under the O_2 proportion of 0% (Fig. 4h) rather than the optimum O_2 proportion (75%) for CH_3CONH_2 synthesis due to the following reason. Under higher concentrations of dissolved O_2, the trapping of $^{\bullet}O_2^{-}$ is more favorable to generate DMPO-$^{\bullet}O_2^{-}$ rather than DMPO-$^{\bullet}NH_2$, which makes it difficult to identify the actual N-intermediate.

Moreover, to accumulate more $^{\bullet}NH_2$ for the successful detection of the DMPO-$^{\bullet}NH_2$, the initial NH_3 concentration is elevated to a higher level (Fig. 4h, 0.32 mol L^{-1}) in comparison with that of the $CH_3{}^{\bullet}CHOH$ detection (Fig. 4b, 0.02 mol L^{-1}), leading to the absence of the signals of DMPO-$CH_3{}^{\bullet}CHOH$ species.

The trapping priority of DMPO is decreased in the order of DMPO-$^{\bullet}O_2^{-}$ > DMPO-$CH_3CH_2O^{\bullet}$ > DMPO-$CH_3{}^{\bullet}CHOH$ > DMPO-$^{\bullet}NH_2$. Hence, the optimized test parameters, i.e., higher NH_3 concentration under 0% of O_2 proportion, are indispensable. Otherwise, the $^{\bullet}NH_2$ cannot be precisely trapped and detected. Based on these results, it is still convincible that h^{+} is the dominant oxidative driving force for the selective NH_3 oxidation into $^{\bullet}NH_2$ radical as the transient intermediate for C-N coupling.

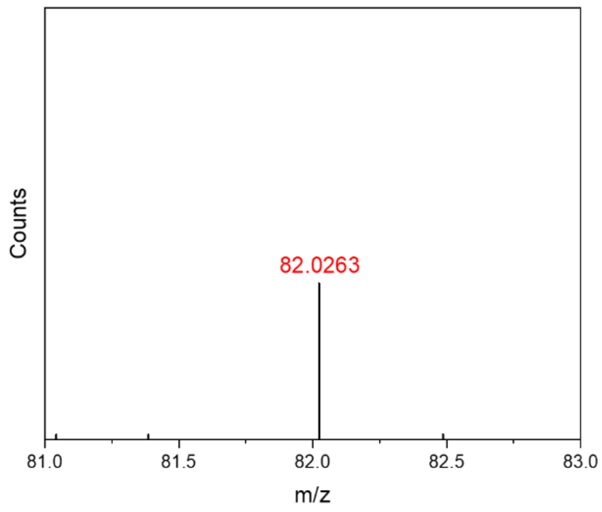

Supplementary Figure 61 | HR-MS results. HR-MS results for the detection of $CH_3CO^{14}NH_2$.

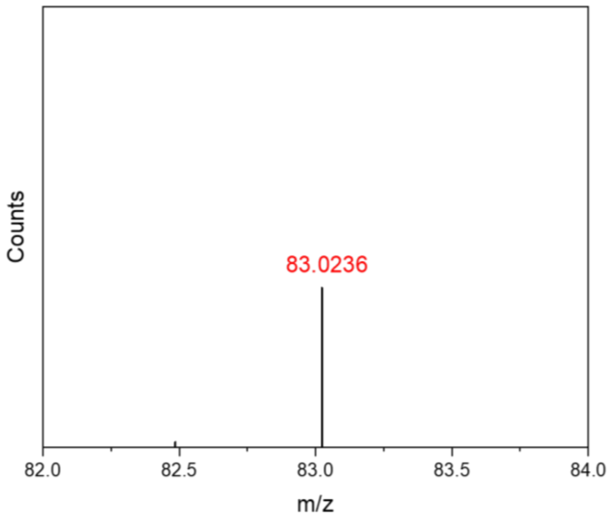

Supplementary Figure 62 | HR-MS results. HR-MS results for the detection of $CH_3CO^{15}NH_2$.

Supplementary Note 25 | m/z of $^{14}N/^{15}N$ labeled acetamide.

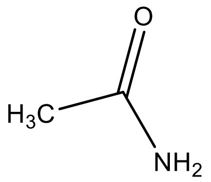

ESI-MS (ESI+): calculated m/z [M+Na] $^+$ for [C_2H_5NaNO] $^+$: 82.0264, found: 82.0263.

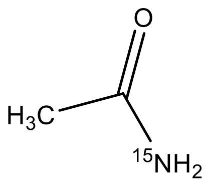

ESI-MS (ESI+): calculated m/z [M+Na] $^+$ for [$C_2H_5Na^{15}NO$] $^+$: 83.0234, found: 82.0236.

Supplementary Note 26 | Materials and chemicals.

The commercial P25 was purchased from Degussa Co. Ltd. $NH_3 \cdot H_2O$ (≥28%), KNO_3 (AR, 99.0%), HCOOH(Chromatographic Pure, CP, ≥98%), CH_3OOH (AR, ≥99.5%), CH_3CHO (standard for GC, ≥99.5%), CH_3CONH_2 (standard for GC, ≥99.5%), urea (AR, ≥99%), $HCONH_2$ (AR, 99%), CD_3CD_2OD (99%), $^{15}NH_4Cl$ (≥98%), CH_3CN (AR, ≥99%), $Bi(NO_3) \cdot 5H_2O$ (≥98%), KCl (99%), KBr (99%), $C_4H_6O_4Sr$ (99%), $K_2H_2Sb_2O_7$ (99%), TEMP (98%), and TEMPO (98%) were purchased from Aladdin Biochemical Technology Co., Ltd.. NH_3 in Ethanol (2 mol/L) was purchased from Shanghai Adamasi Reagents. KNO_2 (Analytical Reagent, AR, 97%) and CH_3COONa (≥99.9% metals basis) were purchased from Shanghai Macklin Biochemical Co., Ltd.. CH_3OH (AR, ≥99.7%) and CH_3CH_2OH (AR, ≥99.7%) were purchased from Shanghai Titan Scientific Co., Ltd.. DMPO (AR) was purchased from DOJINDO LABORATORIES.

Supplementary References

1 Yang, S. et al. Photocatalytic Co-Reduction of N_2 and CO_2 with CeO_2 Catalyst for Urea Synthesis. *Angew. Chem. Int. Ed.* **62**, e202312076, (2023).

2 Shi, C. et al. Nitric Acid-Mediated Artificial Urea Photo-Synthesis With N_2 And CO_2. *Adv. Energy Mater.* **14**, 2400201 (2024).

3 Yuan, M. et al. Host–guest molecular interaction promoted urea electrosynthesis over a precisely designed conductive metal–organic framework. *Energy Environ. Sci.* **15**, 2084-2095.

4 Yang, W., Xiao, L., Dai, W., Mou, S. & Dong, F. Efficient Solar Driven Upgrading of N_2 to Urea Through Photoredox Reactions on Pt Cluster/TiO_2. *Adv. Energy Mater.*, 2303806, (2024).

5 Yang, W. et al. Photocatalytic Formamide Synthesis via Coupling of Electrophilic and Nucleophilic Radicals over Atomically Dispersed Bi Sites. *Angew. Chem. Int. Ed.* **2024**, e202408379.

6 Li, P., Zhao, W., Wang, K., Wang, T. & Zhang, B. Photocatalytic Synthesis of Glycine from Methanol and Nitrate. *Angew. Chem. Int. Ed.* **2024**, e202405370.

7 Li, W. et al. Atomic Ruthenium-Promoted Cadmium Sulfide for Photocatalytic Production of Amino Acids from Biomass Derivatives. *Angew. Chem. Int. Ed.* e202320014 (2024).

8 Liu, Q., Lin, J., Cheng, H., Wei, L. & Wang, F. Simultaneous co-Photocatalytic CO_2 Reduction and Ethanol Oxidation towards Synergistic Acetaldehyde Synthesis. *Angew. Chem. Int. Ed.* **62**, e202218720 (2023).

9 Li, J. & Kornienko, N. Electrochemically driven C-N bond formation from CO_2 and ammonia at the triple-phase boundary. *Chem. Sci.* **13**, 3957-3964, (2022).

10 Kuang, S. et al. Acetamide Electrosynthesis from CO_2 and Nitrite in Water. *Angew. Chem. Int. Ed.* **63**, e202316772 (2024).

11 Meng, N., Huang, Y., Liu, Y., Yu, Y. & Zhang, B. Electrosynthesis of urea from nitrite and CO_2 over oxygen vacancy-rich ZnO porous nanosheets. *Cell Rep. Phys. Sci.* **2**, 100378, (2021).

12 Wang, J. et al. Regulating the Ammonia Oxidation Selectivity via the Quantified Provision of Molecular Oxygen. *ACS Catal.* **13**, 8783-8791, (2023).

13 Hu, Q. et al. Ammonia Electrosynthesis from Nitrate Using a Ruthenium-Copper Cocatalyst System: A Full Concentration Range Study. *J. Am. Chem. Soc.* **146**, 668-676, (2023).

14 Wang, H., Sun, Y. & Dong, F. Insight into the Overlooked Photochemical Decomposition of Atmospheric Surface Nitrates Triggered by Visible Light. *Angew. Chem. Int. Ed.* **61**, e202209201 (2022).

15 Wu, J. et al. Integrated Tandem Electrochemical-chemical-electrochemical Coupling of Biomass and Nitrate to Sustainable Alanine. *Angew. Chem. Int. Ed.* **62**, e202311196 (2023).

16 Li, M. et al. Electrosynthesis of amino acids from NO and α-keto acids using two decoupled flow reactors. *Nat. Catal.* **6**, 906-915, (2023).

17 Dong, X. a. et al. Insights into Dynamic Surface Bromide Sites in $Bi_4O_5Br_2$ for Sustainable N_2 Photofixation. *Angew. Chem. Int. Ed.* **61**, e202200937 (2022).

18 Li, Y. et al. Sequential co-reduction of nitrate and carbon dioxide enables selective urea electrosynthesis. *Nat. Commun.* **15**, 176, (2024).

19 Guo, C. et al. Electrochemical Upgrading of Formic Acid to Formamide via Coupling Nitrite Co-Reduction. *J. Am. Chem. Soc.* **144**, 16006-16011, (2022).
